# Impact of a Spanish for Specific Purposes in Agriculture Curriculum for Animal Science and Veterinary Students on Animal Welfare Knowledge of Spanish-Speaking Livestock Workers

**DOI:** 10.3390/ani15172506

**Published:** 2025-08-26

**Authors:** Allen Jimena Martinez Aguiriano, Leonor Salazar, Silvana Pietrosemoli, Babafela Awosile, Pablo Lamino, Arlene Garcia

**Affiliations:** 1 School of Veterinary Medicine, Texas Tech University, Amarillo, TX 79106, USA; allemart@ttu.edu (A.J.M.A.);; 2 Department of Animal Science, North Carolina State University, Raleigh, NC 27695, USA; 3 Department of Agricultural Education and Communication, University of Florida, Gainesville, FL 32603, USA

**Keywords:** Spanish for Specific Purposes, animal welfare, animal science and veterinary students, farmworkers’ knowledge, communication gaps

## Abstract

This study evaluates the impact that Spanish for Specific Purposes in Agriculture (SSPA) courses taken by animal science and veterinary medicine students have on animal welfare knowledge among Spanish-speaking livestock workers in Texas. The paper describes an internship experience in which animal science and veterinary students delivered animal welfare presentations in Spanish to Spanish-speaking livestock workers while gaining practical skills with a particular animal species. Guided by a One Welfare and Intercultural Communicative Competence framework and comparing survey data collected before and after the student’s intervention, this investigation aimed to identify workers’ improvements in understanding animal welfare issues, assess the effectiveness of communication efforts in the Spanish language, and understand livestock workers’ attitudes towards the welfare training program. The results were examined using a mixed-method approach, integrating quantitative assessments of knowledge with qualitative insights into farmworkers’ perceptions, showing that Spanish competence developed by students through the SSPA curriculum had a positive impact on the self-perceived knowledge of animal welfare practices among Spanish-speaking livestock workers. The most impacted animal welfare areas were acts of abuse and transport.

## Simple Summary

Key operations in the U.S. livestock industry rely on a diverse workforce comprising a significant number of Spanish-speaking workers. However, language communication barriers often arise between farm personnel and animal caretakers given that English-speaking farm owners, managers, veterinarians, and animal scientists, among others, usually have limited proficiency in Spanish, while many workers have insufficient English skills. This scenario entails the likelihood of impacting not only farm operations and husbandry practices in general but can severely affect animal health and welfare. To minimize the ongoing challenges imposed by language barriers, this research aimed at evaluating the impact of a Spanish for Specific Purposes in Agriculture (SSPA) curriculum for English-speaking animal science and veterinary students on the knowledge level of Spanish-speaking livestock workers in terms of animal welfare. Upon completion of an SSPA program, the students enrolled in farm internships involving swine, poultry, beef and dairy cattle with the purpose of improving language skills through their interactions with livestock workers and delivering animal welfare presentations both in Spanish. The results of pre- and post-surveys showed that Spanish competence developed by students in the framework of the SSPA curriculum positively impacted the self-perceived knowledge of animal welfare among the Spanish-speaking livestock workers.

## 1. Introduction

### 1.1. Communication Gaps in Agriculture and Animal Welfare

Animal welfare has increasingly become a key element of animal production. Although many definitions of welfare have been proposed in the livestock industry throughout the years, a single unified concept is still not available since it is extremely difficult to integrate multiple viewpoints that fit all the situations, environmental circumstances, management practices, personal and corporative interests and goals, among other factors. To talk about welfare in a comprehensive and informed manner, it is paramount to consider that the term is firmly rooted in ethical principles that stem from the fact that animals are sentient beings, which implies that they should be raised, transported, and slaughtered as humanely as possible. For the purpose of this research, animal welfare was holistically conceived as the positive state of an individual resulting from adequate practices that consider aspects related to physiology, health, emotional state, and animal behavior, which in turn contribute to the generation of responsible and sustainable production systems. This involves providing stress-free environment and care, freedom to express natural behavior, humane handling, adequate management and nutrition, disease prevention and treatment, proper housing, and even humane euthanasia [1].

Animal welfare is a vital part of the U.S agricultural economy including swine, poultry, beef and dairy cattle production. These industries depend heavily on a diverse workforce that includes many Spanish-speaking farmworkers who are critical in daily operations [2,3,4,5]. However, language communication barriers are a persistent challenge on farms [2,6]. Many farmworkers have limited English proficiency, and most farm owners and veterinarians do not speak Spanish [7,8]. These communication gaps can significantly affect farm management efficiency and the welfare of the animals under their care [6,9]. Additionally, limited English proficiency among Spanish-speaking workers interferes with their career advancement opportunities and prevents them from taking on leadership roles [10].

Effective communication between farm owners, veterinarians, and workers is essential for maintaining high animal health and welfare standards. Research has shown that clear communication can improve handling techniques, reduce animal stress, and prevent behavioral issues [11,12]. Training delivered in a language that workers understand can improve animal care and worker performance [13]. Studies have shown that Spanish-language instruction on dairy farms has strengthened the relationship between veterinary professionals and farmworkers [14,15]. In contrast, communication failure can lead to miscommunication, delayed interventions, and improper care, compromising animal health outcomes and reducing safety [16,17,18,19,20].

This is not just a U.S. issue. International research, including studies on Danish farms with multicultural workforces, highlights how language and cultural barriers can isolate foreign workers, prevent meaningful integration, and negatively impact animal welfare [9,21]. These deficiencies extend beyond language; they also reflect a lack of cultural awareness and limited training [21].

### 1.2. The Role of Veterinary and Animal Science Professionals in Livestock Production and the Need for Targeted Language Training

Animal scientists and veterinarians are key to animal welfare. Their work goes beyond clinical and animal care; they are educators, advocates, and bridges between science and the people caring for animals on a daily basis [22]. Despite increasing exposure to limited English proficient (LEP) Spanish-speaking livestock workers, only a minority of U.S. veterinary professionals are equipped with the tools to communicate effectively across cultures or languages.

Effective communication is also crucial in veterinary clinical practice. Research indicates that veterinarians who can communicate effectively have better client satisfaction and treatment adherence, leading to improved animal care [23,24].

Veterinary professionals increasingly work with limited English proficiency (LEP) clients or farmworkers, especially in states with large Spanish-speaking populations. However, only a minority feel equipped to engage with these clients after gaining experience at a practice or shelter that serves LEP Spanish-speaking individuals [25]. Surveys reveal that just 8% of small animal practitioners can communicate in Spanish, while many express a desire for Spanish language training or to work with bilingual staff. The disconnect between the needs of Spanish-speaking populations and the linguistic capabilities of veterinary and animal science professionals is widening [25]. Research also shows that although 63% of students acknowledge the significance of communicating with LEP Spanish-speaking clients in Veterinary Medicine, only 55% feel confident in their technical communication abilities, and 32% of those with interpreting experience do not consider themselves conversant in Spanish [25]. Moreover, only 16.4% of veterinary students consider themselves fluent in Spanish, and fewer than 8% feel sufficiently prepared to translate medical information to Spanish-speaking clients [26].

Feedback from previous Spanish program initiatives highlights several challenges. Some students found the pace of Spanish courses too fast, leading to overwhelming feelings, especially for those with little or no Spanish experience [27]. This emphasizes the necessity for adaptable and well-organized course designs that address students’ varied learning requirements [27]. This social and purpose-specific Spanish proficiency gap underscores the need for improved and specific training. It reveals the limitations of a one-size-fits-all approach to language instruction and emphasizes the need for more flexible and structured course designs [27].

### 1.3. Spanish for Specific Purposes in Agriculture (SSPA)

Our research team addressed the gap described earlier by developing and implementing a Spanish for Specific Purposes in Agriculture (SSPA) program/curriculum that focused on specialized terminology and on farm daily live context since, general Spanish classes do not cover the vocabulary or real-world scenarios that farm owners, animal scientists, and veterinarians encounter in daily activities involving animal care. The novelty of this SSPA curriculum relies on rigorous scientific findings involving specific communication needs reported by veterinarians, animal scientists, farm owners, farm managers, animal nutritionists, agribusiness consultants, farm trainers, and professors [28]. The SSPA curriculum was designed with those specific needs in mind and therefore incorporated activities to encourage students to communicate with livestock workers regarding animal health, welfare, biosecurity, and other pertinent agricultural topics in a practical and culturally relevant way. Another novel aspect of this SSPA program is that it incorporates farm internships as a mean for providing more opportunities to students to expand their Spanish competences and skills as they interact with and deliver animal welfare presentations to native Spanish-speakers. In other industries, similar training approaches have proven successful [29,30,31]. Hybrid courses have led to gains in both knowledge and behavior. For example, face-to-face instruction combined with online components significantly increased pesticide safety knowledge and practices [32]. Similarly, a safety and health training program for dairy workers, delivered in Spanish, led to statistically significant gains in knowledge and farmworker behavior changes, reducing workplace hazards [33,34]. These examples demonstrate that culturally and linguistically specific training can lead to meaningful behavioral changes. This is especially relevant in animal production, where the consequences of communication gaps can directly impact both human and animal welfare.

It is essential to recognize that poor animal welfare on farms is not always the result of cruelty or neglect. More often, it arises from limited awareness or critical gaps in training and education [35]. In many cases, livestock workers continue doing what they were taught or what they saw, unaware that other alternatives exist. This is where humane education becomes transformative, since it is capable of transmitting knowledge and reshaping habits and attitudes [36].

However, for this education to be truly effective, it must account for more than just language; it must be culturally appropriate. In agricultural environments, how Spanish-speaking farmworkers perceive, question, and apply information is deeply influenced by their cultural norms [37,38]. Simple cultural differences, such as personal space, tone, or how questions are asked, can significantly affect how training is received [39,40]. Thus, meaningful communication in this context requires linguistic proficiency and cultural empathy.

We have previously identified specific language needs for animal professionals interacting with Spanish-speaking animal caretakers, ensuring the relevance and practicality of the intervention [28]. This led to the development and implementation of three SSPA courses within the veterinary medicine and animal science curriculum, addressing the identified needs [41]. This article reports the results of an internship program grounded in experiential learning theory that emphasizes experience as a critical source of learning [42]. In turn, it aligns with the communicative learning approach, which prioritizes language as a tool for meaningful interaction [43,44]. Constructivist principles further guided the design, framing learning as an active process in which knowledge is built through interaction with the environment and peers [45].

This study explores how English-speaking animal sciences and veterinary students put into practice their previously built Spanish language skills in a practical on-farm environment by delivering animal welfare presentations in Spanish to Spanish-speaking livestock workers. These presentations were based on scientific principles and topics relevant to each species’ animal welfare audit standards (swine, poultry, beef and dairy cattle). The hands-on experience allowed students to enhance their Spanish and communication abilities while providing essential information in Spanish to farmworkers to increase awareness about animal welfare topics and encourage positive changes on farms. This study adopts an explanatory mixed methods approach, initiating with the collection of quantitative survey data to evaluate how farmworkers’ animal welfare knowledge shifts after the training intervention. To enhance our understanding of participants’ views on the training’s relevance, clarity, and delivery, these data were supplemented by qualitative feedback from open-ended question responses. This method facilitated a more accurate interpretation of results, linking statistical outcomes with livestock workers’ real experiences and insights, thus offering a comprehensive evaluation of the SSPA’s impact. This integrated approach not only aids in developing language skills but also aligns with the broader mission of improving animal welfare within the agricultural sector, reflecting a comprehensive One Health perspective.

This study aims to address several important questions: What happens when students who have completed the SSPA courses join the workforce? Can they effectively communicate topics in Spanish to livestock workers and participate in a linguistically and culturally diverse farming environment? More importantly, does this training improve Spanish-speaking farmworkers’ comprehension of animal welfare? What elements contribute to the success of this intervention, and which aspects could be enhanced? Can the involvement of students influence the livestock workers’ understanding of animal welfare topics? Using a mixed-methods approach, we evaluated pre- and post-intervention knowledge, gathered qualitative feedback, and analyzed statistical relationships between participant characteristics and outcomes. Our goal was to determine the impact of student SSPA competence and welfare training sessions on the knowledge of animal health and welfare among Spanish-speaking farmworkers. This study contributes to the existing literature on cross-cultural communication, animal welfare education, and hands-on veterinary training in livestock farms by introducing a scalable intervention model to bridge the language gap and optimize livestock care.

## 2. Methodology

This study was approved by the Human Research Protection Program at Texas Tech University (IRB2021-250).

### 2.1. Study Design

This study was designed to evaluate the effectiveness of an SSPA curriculum in improving the knowledge of Hispanic farmworkers about animal health and welfare. Animal science and Veterinary students conducted educational sessions on farms addressed to Spanish-speaking farmworkers across swine, poultry, beef and dairy cattle production systems. A quasi-experimental pretest-posttest design without a control group was utilized. An explanatory sequential mixed-methods approach was implemented. Quantitative data were gathered and analyzed through pre- and post-tests. In addition, qualitative data were collected in the post-test through 3 open-ended questions that served to examine further livestock workers’ perceptions, preferences, and suggestions for improving the welfare training program. The qualitative insights helped to better interpret and contextualize specific quantitative findings, offering a more well-rounded understanding of the intervention’s impact.

### 2.2. Design of Data Collection Instruments

Pre and post surveys were drafted to collect data pertaining workers’ self-perceived knowledge on animal welfare. The instruments consisted of the same anonymous survey comprising 25 closed-ended dichotomous (yes/no) questions representing 12 key animal welfare topics, featuring two questions per topic, except for euthanasia, which had three questions. Surveys were organized into three sections: a: demographic and general information about the livestock workers (Appendix A), and b: questions evaluating knowledge on the 12 core animal welfare topics (Appendix B and Appendix C). The post-survey also included c. items to collect feedback on the training provided (Appendix D). To ensure reliability, the instruments were validated by five subject matter experts including professionals from both academia and the industry. The reviewers conducted an evaluation of the questions to ensure clarity, accuracy of content, cultural appropriateness, and alignment with established farm practices. The feedback they provided guided targeted minor revisions, which were subsequently incorporated into the final version of the instrument.

### 2.3. Design of Training Tools

Twelve PowerPoint presentations were developed for each livestock farm production system. These presentations were crafted to meet established audit standards, such as the Common Swine Industry Audit [46] and the American Humane Certified [47]. The content was organized around 12 core animal welfare topics: acts of abuse, euthanasia, animal handling, biosecurity, animal health, records, feeding and water intake, lameness, transport, housing, mortality, and behavior. Farms lacking presentation equipment were provided with a data projector. Presentations were delivered during the farm’s lunch breaks.

### 2.4. Participants

Participants were recruited using a convenience sampling method, relying on the availability of livestock workers and established agreements between the researchers and farms. The intervention took place on eight farms reaching four livestock sectors: swine, poultry, beef and dairy cattle, located in the following regions of Texas: the Panhandle (Dalhart, Perryton), West Texas (Idalou), and North-Central Texas (Dublin, Stephenville). This diverse geographic distribution facilitated the inclusion of various production systems and worker profiles. A total of 79 Spanish-speaking farmworkers completed both pre- and post-training surveys. Participants were required to attend 12 training sessions to be eligible for the study.

Post-survey data collection was organized to ensure that all participants from the previous survey were present. Survey respondents were selected by the farm managers, who employed different selection criteria: some selected only experienced workers, while others permitted all staff members to participate, introducing potential variability and selection bias. Survey completion was voluntary, and instructions were delivered verbally in Spanish before data collection. The purpose and procedures of the training sessions were also explained.

### 2.5. Student Training and Internship Assignment

After completing three SSPA courses, students were assigned to farms according to their species-specific interests. They were placed on a farm in pairs, and a total of 12 presentations were conducted, with each student giving 6 presentations on the core animal welfare topics previously mentioned. Before starting their internships on the farms, students engaged in oral communication practice sessions and received educational resources (PowerPoint presentations) previously developed by the research team.

### 2.6. Student Interventions

Throughout six weeks, students worked on the farms while delivering interactive presentations to workers on a weekly basis. The researchers followed up with students biweekly to evaluate progress, answer questions, provide feedback, and suggest ways to improve. The content of these presentations was tailored to each animal species (Appendix E, Appendix F, Appendix G and Appendix H).

### 2.7. Pre- and Post-Survey Administration

Before the student presentations, an anonymous survey (Appendix A, Appendix B, Appendix C and Appendix D) was administered to assess the initial understanding of animal welfare practices among participating livestock workers and to collect demographic data. A total of 111 pre-surveys were collected from eight participating farms. The survey was conducted in Spanish and read aloud to respondents to enhance understanding, especially for livestock workers with low literacy levels. In some cases, verbal examples were provided to clarify the questions. Moreover, managers were requested not to be present during the surveys to minimize response bias. The anonymity of both surveys contributed to reducing any potential social desirability bias. Livestock workers were informed that their answers would remain confidential and not be shared with their employers. They were assured their responses would not lead to any judgment or negative consequences concerning their jobs, and that all answers would be used only for research purposes.

Responses were collected using a paper-and-pencil instrument. Following the students’ final presentation, the same livestock workers completed an anonymous post-survey identical to the one administered before the intervention but included additional feedback questions (Appendix D). This allowed for a direct comparison of pre- and post-intervention knowledge levels. In addition, quantitative and qualitative feedback was collected from the livestock workers regarding the usefulness of the training, student communication skills, observed improvements in workplace practices, and overall effectiveness of the students’ presentations.

### 2.8. Data Analysis

Survey responses were transcribed and translated into English. Microsoft Excel for Microsoft 365 MSO (Version 240) was utilized to categorize and organize this data. Quantitative data were analyzed using R statistical software (Version 4.3.2). Descriptive and inferential analyses assessed the effectiveness of the SSPA curriculum on livestock workers’ knowledge concerning welfare.

The McNemar Test and the Wilcoxon Signed-Rank Test were utilized for inferential analysis. The McNemar test assessed pre- and post-survey results on categorical items, while descriptive statistics helped analyze demographic trends. The Wilcoxon Signed-Rank Test was used on ordinal data (topic scores ranging from 0 to 1) to determine if the median difference in knowledge scores from pre- to post-training was statistically significant. This non-parametric test was suitable because of the data’s non-normal distribution and the paired design. Additionally, heatmaps were created to illustrate knowledge gains across different farm types and education levels.

### 2.9. Evaluation of Knowledge Gain by Sector

The improvement in knowledge among livestock workers was evaluated by comparing survey scores from before and after training across the four livestock sectors. For each sector, the total number of affirmative responses from each worker was added up, and a percentage score was computed based on the maximum possible correct answers (number of questions multiplied by the number of participants). The percentage difference between the average post-training and pre-training scores determined knowledge gain.

### 2.10. Qualitative Thematic Analysis and Demographic Comparison

To examine Spanish-speaking farmworkers’ perceptions of the training program and communication challenges, a qualitative thematic analysis was conducted using Clarke and Braun’s six-phase framework [48]. This comprehensive approach allowed for an in-depth exploration of participants’ perspectives, capturing insights into the training experience and suggestions. The analysis was based on responses to two open-ended post-survey questions:

1. “Do you feel there was an improvement in your knowledge? Why?”
2. “Can you offer us any suggestions or comments about the training?”

All responses were initially written in Spanish and translated into English, preserving the original intent and meaning. Following grounded-theory principles, a codebook was developed inductively from the data [49]. Open coding was used to analyze the responses line by line, identifying emergent concepts and patterns [50].

A double coding process was employed to enhance reliability. Two researchers independently coded the data. Then, intercoder reliability was evaluated using Cohen’s Kappa with the Statistical Package for the Social Sciences (SPSS), version 29.0 (IBM Corp., Armonk, NY, USA) for each question [51,52]. The resulting kappa values were interpreted according to Landis and Koch [53], slight agreement (0.01–0.20), fair (0.21–0.40), moderate (0.41–0.60), substantial (0.61–0.80), and almost perfect (0.81–1.00). Following this, the coders met to discuss discrepancies by reviewing a subset of responses (three quotes from the first question and four from the second), reaching complete agreement and refining the codebook accordingly.

To explore whether feedback varied by background, coded themes were cross-tabulated with demographic variables (e.g., gender, education, and years of experience), and chi-square tests were performed with the Statistical Package for the Social Sciences (SPSS), version 29.0 (IBM Corp., Armonk, NY, USA) to identify statistically significant associations.

### 2.11. Multivariable Logistic Regression Analysis: Training Difficulty Perception and Course Satisfaction

Two distinct multivariable logistic regression analyses were conducted to assess the relationships between demographic factors (gender, age, years of experience, and education level) and participants’ (1) perception of training difficulty and (2) satisfaction with the training program. Each outcome variable, perception, and satisfaction level were analyzed as binary variables. All demographic predictors were incorporated into the models simultaneously. Odds ratios were calculated, with 95% confidence intervals, and *p*-values for each predictor, with statistical significance as *p* < 0.05.

The dependent variable for difficulty perception was measured using a 5-point Likert-type scale, ranging from “Not difficult at all” to “Extremely difficult.” Similarly, the dependent variable for satisfaction was quantified using a 5-point Likert-type scale, ranging from “not satisfied at all” to “extremely satisfied.”

### 2.12. Student-Instructor Performance Across Livestock Sectors

The effectiveness of the students’ training sessions across various livestock sectors was evaluated based on eight instructional criteria: topic knowledge, language proficiency, pronunciation, fluency, intonation, audience interaction, confidence, voice and volume, and time management. Four performance indicators were used: punctuality, task engagement, willingness to communicate in Spanish, and overall performance. Bilingual farm managers or site supervisors completed a post-training evaluation for each student, using a standardized rubric that employs a 1–4 scale: deficient (1), fair (2), good (3), and excellent (4). A descriptive analysis was conducted. The researchers developed this evaluation and asked for the collaboration of a bilingual farm supervisor to assess student communication, delivery, and professionalism during training sessions.

## 3. Results

### 3.1. Farm Participation

79 post-surveys were completed and matched with the pre-surveys: The effective response rate was 71%.

### 3.2. Demographic Profile of Participants (Livestock Workers Profile)

Seventy-nine Spanish-speaking farmworkers took part in the study. The demographic details are as follows (Table 1):

Age: Participants’ ages ranged from 18 to 56. Most were in the 18–35 range (35 individuals, 44.3%). The 36–55 age group included 19 participants (24.1%), and only 3 participants (3.8%) were 56 or older. The average age among participants was 36 years, with the youngest being 18 and the oldest 56. These results indicate a young workforce, with many workers in their early to mid-career stages.

Educational Background: Farmworkers’ educational levels were grouped into low and higher education. Basic education: This category includes livestock workers who completed elementary school and high school education. Forty-two livestock workers (53.2% of the sample) fell into this category. Higher education: This group included livestock workers who completed technical or university-level education. Thirty-seven workers (46.8% of the sample) were classified under this category.

These results indicate that slightly more than half of the participants (53.2%) had completed basic education, while half, also 46.8%, had attained some form of higher education.

Level of Experience: Farmworkers’ years of experience were categorized into six groups: less than 1 year (11 workers, 13.9% of the sample), 1 to 4 years (35 workers, 44.3%), 5 to 10 years (17 workers, 21.5%), 11 to 15 years (6 workers, 7.6%), 16 to 20 years (8 workers, 10.1%), and more than 20 years (2 workers, 2.5%). Most participants (58.2%) had less than 5 years of experience, while a smaller proportion (20.2%) had more than 10 years of experience. This distribution suggests that most livestock workers were new to the field, with only a few having extensive experience. The average years of experience was 6 years, with a minimum of 2 months and a maximum of 26 years.

Table 1 illustrates demographic differences among livestock sectors, especially regarding gender distribution, education levels, and migration status. These factors can impact baseline knowledge (pre-survey) and the ability to respond to training (post-survey).

Cronbach’s alpha was calculated to evaluate the internal consistency of the survey. The analysis indicated good internal consistency, with a Cronbach’s alpha of 0.872 for pre-survey (standardized alpha = 0.880) and 0.848 for post-survey, demonstrating that the 25 survey items reliably measured the same underlying construct.

### 3.3. Pre- and Post-Training Knowledge Assessment

A McNemar Test was conducted after student intervention, revealing that Question 4: “I know what constitutes an act of animal abuse”-Acts of abuse (0.0076) and Question 19: “I know which animals are fit for transport according to their health status” -Transport (0.0076) differed significantly (*p* < 0.05), showing an improvement between pre- and post-knowledge. While other topics showed improvement in raw scores, the differences were not statistically significant at the 0.05 level (Table 2).

**Figure 1:**
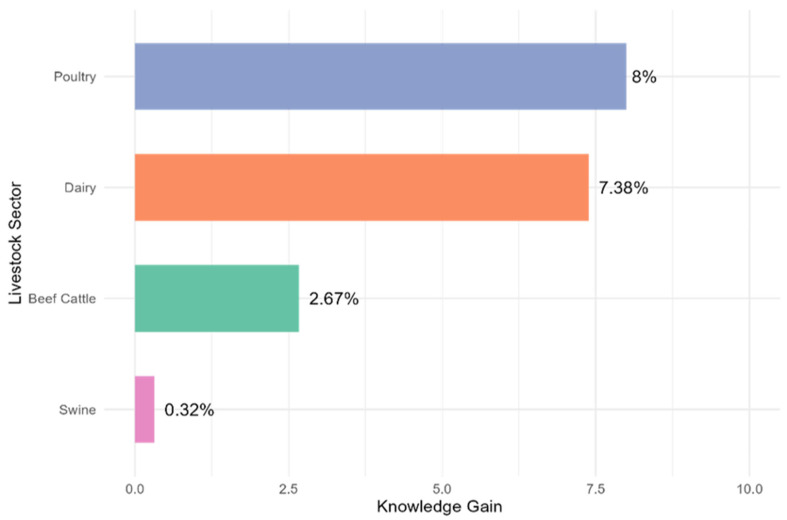
Livestock Workers’ Animal Welfare Knowledge Gain by the Livestock Sector.

The overall average pre-survey score was 0.86, indicating that participants already had approximately 86.2% of the knowledge before the training. After completing the training, the average score increased to 0.936, 93.6% (7.4% improvement). These results reflect a positive change in worker knowledge following the SSPA-based intervention (Figure 1). Poultry workers demonstrated the highest gain of knowledge (+8.0%), followed closely by dairy workers (+7.4%). Beef cattle showed moderate improvement (+2.7%), while swine workers exhibited a marginal gain (+0.3%), reflecting a ceiling effect due to high baseline knowledge. These findings emphasize the need for tailoring training interventions to sector-specific needs and initial worker knowledge levels to maximize educational outcomes.

### 3.4. Differences by Livestock Sector-Based Knowledge

**Figure 2:**
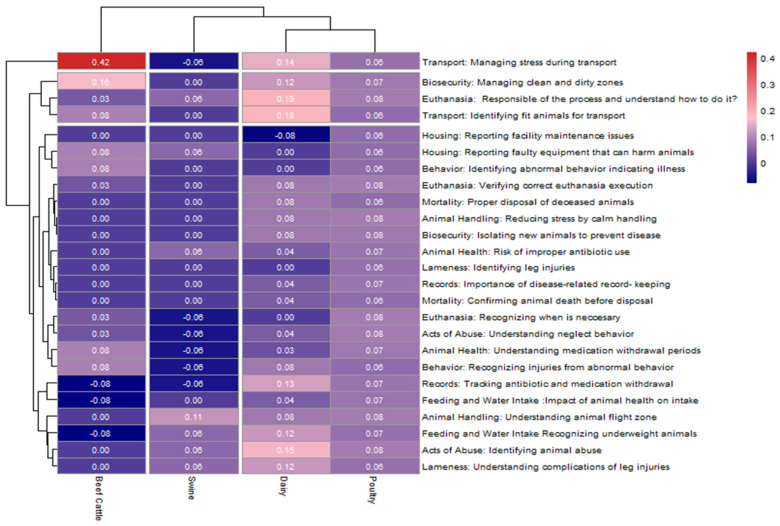
Livestock Workers’ Knowledge Gain by Livestock Sector and Animal Welfare Topic.

A topic knowledge-level heatmap analysis (Figure 2) showed the variation in knowledge gains by livestock sector. Beef cattle workers demonstrated the most significant individual topic gain with an increase of +0.42 in knowledge related to transport stress management, followed by +0.10 in biosecurity protocols.

Dairy livestock workers showed moderate improvements, especially in Transport (+0.14 and +0.18), euthanasia (+0.19), acts of abuse (0.15), and feeding and water intake (+0.12). Poultry workers benefited from all the topics, with no significant negative changes.

In contrast, swine livestock workers exhibited minimal or slightly negative gains across most topics. Gains were limited, and in some cases, topic scores remained unchanged or decreased slightly.

### 3.5. Knowledge Differences by Education Level

**Figure 3:**
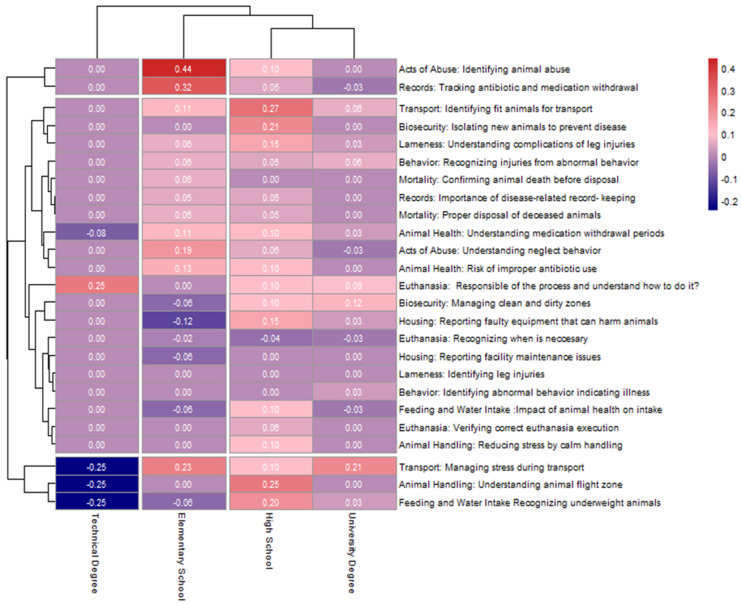
Livestock Workers’ Knowledge Gain by Animal Welfare Topic and Educational Background.

A topic heatmap (Figure 3) revealed that livestock workers with an elementary school background exhibited the most substantial knowledge gains in key areas such as identifying acts of abuse (+0.44), record keeping for antibiotics (+0.32), and managing stress during transport (+0.23). These results suggest that the training effectively addressed foundational knowledge gaps among participants at lower levels of education. Participants with high school and university-level education showed moderate improvement across a broader range of topics, including biosecurity, animal health, and transport management. This suggests that while they may have entered the training with greater baseline knowledge, the content still reinforces their understanding of animal welfare topics. In contrast, livestock workers with technical degrees showed minor negative differences across multiple topics. These included areas related to stress management during transport, animal handling, and identification of underweight animals. The clustering structure in the heatmap grouped similar knowledge gain patterns across education levels. Topics such as euthanasia procedures, biosecurity, and transport appeared in closely aligned clusters, indicating consistent performance across groups. Meanwhile, areas showing minimal or negative change among technical degree holders also clustered together.

### 3.6. Worker Level of Learning Outcomes

An analysis was conducted to assess individual-level learning outcomes by topic. Based on changes between pre- and post-surveys’ responses, each participant was classified as “Improved,” “Worsened,” or “No Change.” The topics with the highest proportion of workers showing improvement were transport (21.5%), animal health (15.2%), and acts of abuse (13.9%). Most participants demonstrated no change, suggesting a strong baseline understanding across several welfare domains. Only a small percentage of responses (2.8%) indicated a decline in topic-specific knowledge.

### 3.7. Satisfaction with the Training and Perception of Difficulty

The descriptive analysis of satisfaction levels show that most participants viewed the training favorably. Specifically, only two livestock workers (2.5%) reported being “Not satisfied at all,” 6 of them (7.6%) expressed being “Slightly satisfied”, 25 (31.6%) indicated they were “Satisfied,” 31 (39.2%) were “Very satisfied,” while 15 (19.0%) reported being “Extremely satisfied.” Regarding perceived difficulty, most participants believed the training was not difficult. Fifty-eight livestock workers (73.41%) considered the training sessions “Not difficult at all,” 18 workers (22.78%) felt the training “slightly difficult,” and only three found the training “very difficult” (3.79%).

Two multivariable logistic regression analyses were conducted to explore whether individual characteristics influenced these perceptions, one for satisfaction and one for perceived training difficulty. However, none of the demographic variables (gender, age, years of experience, education level, and migratory status) were statistically significant predictors of either perceived training difficulty. Across all models, *p*-values exceeded 0.5, suggesting that participant demographic characteristics did not strongly influence this study’s perceptions of training difficulty and satisfaction.

### 3.8. Perceived Knowledge Gains (Quantitative/Qualitative Analysis)

On the quantitative side, responses to the question: “*Do you feel there was any improvement in your knowledge*?” showed that 61 out of 79 livestock workers (77.2%) reported an improvement following the training. In contrast, 12 (15.2%) indicated no improvement, and 6 (7.6%) did not respond to the yes-or-no question. When asked if the training made them feel more aware of specific topics in their daily work, 64 workers (81.0%) affirmed, 10 (12.7%) reported no difference, and 5 (6.3%) did not respond.

**Figure 4:**
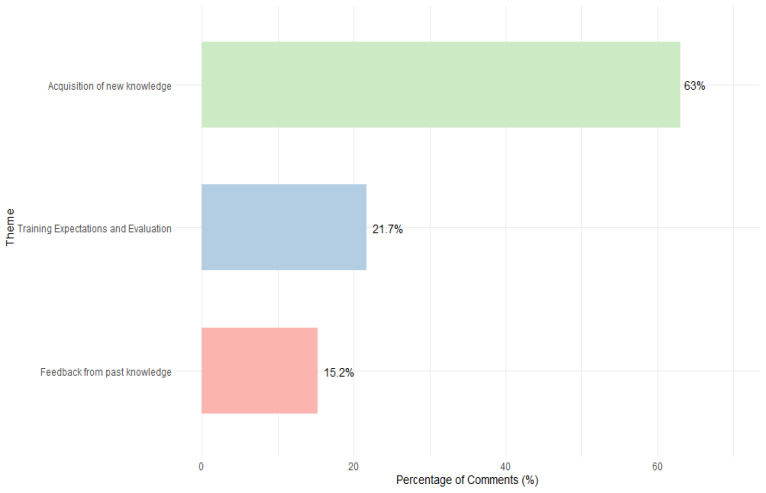
Distribution of Themes Derived from Livestock Workers’ Self-Reported Knowledge Improvement.

A qualitative analysis was performed through thematic analysis on 46 open-ended responses to the follow-up question: *Why do you or don’t you feel there was an improvement?* The results reveal three primary themes reflecting livestock workers’ self-assessed knowledge growth: (1) Acquisition of new knowledge (63.0%), (2) Training expectations and evaluation (21.7%), and (3) Feedback from past knowledge (15.2%). Refer to Figure 4.

#### 3.8.1. Theme 1: Acquisition of New Knowledge

This was the most prominent theme (63.04% *n* = 29), highlighting how participants recognized learning new concepts or gaining clarity on previously misunderstood information.

Subtheme 1: Learning New Content: A significant number of livestock workers (50%, *n* = 23) reported gaining valuable insights during the training sessions, demonstrating the effectiveness of the content. One participant stated: “*We learned some stuff I did not know before*.” (P7), emphasizing the information’s relevance and novelty. Additional remarks included: “*I learned something else about biosecurity*” (P66), “*I learned new stuff and I have been reminded of other stuff*” (P8), “*I learned a lot of new concepts*” (P11), “*I did not know what the animal flight zone was, but now I know*” (P67), “*Now, I know more about animal behavior*” (P13), and “*I learned more about poultry health*” (P63).

Subtheme 2: Clarification of Previous Doubts: A few comments (13.04%, *n* = 6) suggested that the training provided new insights and cleared up previously confusing or misunderstood concepts. For instance, a farmworker stated, “*I used to have doubts before, now I understand*” (P59), highlighting how the sessions resolved lingering questions and deepened their understanding of animal care and handling practices. Other remarks included, “*I have clarity on some of the doubts I had about animals*” (P61) and “*information about the farm can be expanded*” (P15).

These findings demonstrate that the sessions served both as an introduction for some and a clarification tool for others, suggesting that the training effectively addressed varying baseline levels of knowledge.

#### 3.8.2. Theme 2: Training Expectations and Evaluation

This theme reflects farmworkers’ suggestions and preferences regarding content delivery, depth, and trainer characteristics (21.8% *n* = 10).

Subtheme 1: Specific Content Required: Some participants (8.70%, *n* = 4) expressed a desire for more in-depth content that focuses on species-specific topics, particularly concerning animal behavior. For example, workers noted the importance of “*learning a little bit more about animal behavior*” (P9), while others emphasized the need for a “*deeper topic*” (P49).

Subtheme 2: Preferences for Training: A smaller portion of farmworkers (4.35%, *n* = 2) noted that they learn better when trained by individuals they could relate to. One participant remarked, “*I learned better with someone my age*” (P64), indicating that shared identity factors, such as age, can boost learning engagement.

Subtheme 3: More practical content: Another 8.70% of participants (*n* = 4) strongly highlighted the importance of hands-on learning. A common sentiment was the desire to “*incorporate more practical exercises into the training*” (P-55) and to apply the knowledge gained directly in the workplace. Additional comments included, “*More explanations*” (P44) and “*the content was too basic; we need specific practical topics*” (P17). This indicates adult learners’ preference for experiential and visual methods, emphasizing the necessity of integrating demonstrations in future training sessions. These comments reinforce the importance of relevant content delivered in a relatable and applied manner, particularly in vocational contexts like livestock farms.

#### 3.8.3. Theme 3: Feedback from Prior Knowledge

Several participants (15.2%, *n* = 7) indicated that the training reaffirmed existing knowledge. This group perceived the sessions as not merely adding information but as a beneficial refresher. Statements like “*It was a reinforcement of technical knowledge*” (P46), “*I revisited topics from college/university*” (P12), and “*reaffirm knowledge*” (P24) demonstrate how the program fulfilled two roles: imparting updated content to some while reinforcing prior knowledge for others. This highlights the necessity for flexible training methods that cater to learners at various educational stages (Table 3).

### 3.9. General Suggestions and Comments from the Workers About the Training

An open-ended question in the post survey invited participants to share some general suggestions or feedback regarding the training experience. Of the 79 livestock workers, 48 provided comments. The thematic analysis revealed four main themes: (1) Perceptions of training delivery, (2) Requests for more training, (3) Perceptions of training content, and (4) General satisfaction without suggestions.

#### 3.9.1. Theme 1: Perceptions of Training Delivery

This theme is mentioned by 33% of the participants (*n* = 16). It captures farmworkers’ reflections on how the training was delivered, including presentation skills, organization, and audience engagement. While most acknowledged the value of the training content, the quality of delivery significantly influenced the learning experience.

Subtheme 1: Trainer preparation and delivery: Reported by 12.50% of participants (*n* = 6), some farmworkers expressed concerns about the students’ presentation skills and overall preparedness. One farmworker noted, “*They should prepare before presenting. The topics were good, but they did not know how to explain them*” (P26), “*understanding the topic completely before the presentation is important*” (P75), “*be more professional during the trainings*” (P79), “*have more knowledge*” (P28), which reflects the need for stronger communication, presentation, and preparation training among students.

Subtheme 2: Training time and content structure improvement: Highlighted by 10.42% of the participants (*n* = 5), better organization and clearer training schedules are needed. One farmworker recommended “*Set a specific schedule for the training and cover all the important points in farm management*” (P29), “*more time for training*” (P18), this emphasized the importance of assigning enough time and a fixed schedule to deliver specific content of interest which might contribute to better knowledge retention.

Subtheme 3: Audience interaction and engagement: Also noted by 10.42% of participants (*n* = 5), the value of interactive instruction was highlighted; participants noted the need for improvement. One participant remarked, “*Students should engage more actively by asking questions to the audience and be less nervous*” (P42) and “*the students should be able to use their own words to interpret the content while delivering the presentations*” (P44), pointing to a desire for a more dynamic and confident approach to training.

#### 3.9.2. Theme 2: Request for More Training

This theme was mentioned by 29.17% of participants (*n* = 14), highlighting livestock workers’ interest in ongoing education and enhancing animal welfare practices. Many respondents expressed a desire for additional opportunities to strengthen or broaden their skills. One farmworker stated, “*It was good, and it would be even better with follow-up to improve animal handling*” (P56), “*more training about medications of the sow and piglets, and how to identify diseases*” (P32), and “*more training, ask us what we know and then train us on something new*” (P43), emphasizing the importance of continuous learning and a topic selection that aligns with their needs and interests.

#### 3.9.3. Theme 3: Perceptions of Training Content

This theme was emphasized by 20.83% of participants (*n* = 10), reflecting feedback on the training materials’ quality, clarity, and relevance. While many appreciated the content, others requested more in-depth explanations and applied learning opportunities.

Subtheme 1: Practical hands-on content: Farmworkers emphasized the need for additional demonstrations, with 12.50% of participants (*n* = 6) highlighting this matter. One farmworker commented, “*More educational materials and more live demonstrations*” (P10), and “*more practical and more statistics*” (P9), suggesting that experiential training would be better to support comprehension and application on the job.

Subtheme 2: Content depth and technical complexity: A smaller group, 8.33% of participants (*n* = 4), expressed a desire for more advanced content. Some participants required more advanced or technically detailed information. Comments included: “*Approach the training with more technical or comprehensive situations*” (P46) and “*the training should go deeper, for example: teach us about prolapses, why they happen, and sow physiology because this will help us better understand when we work with the sows*” (P34). These indicate that while the foundational material was beneficial, it occasionally fell short in depth.

#### 3.9.4. Theme 4: General Satisfaction Without Suggestions

This theme was mentioned by 16.67% of participants (*n* = 8) who expressed satisfaction with the training without offering critiques. Comments like “*everything was excellent*” (P8) and “*it was great and will be better, after giving a follow-up to improve animal care constantly*” (P56) represent a strong positive response. These remarks suggest that the training met or exceeded expectations for some without the need for changes.

A chi-square test of independence was performed to investigate possible significant associations between the demographic characteristics of livestock workers (age, gender, education level, and years of experience) and their answers to the two open-ended questions. The findings revealed no statistically significant differences, indicating that perceptions and qualitative feedback remained consistent among demographic groups.

**Figure 5:**
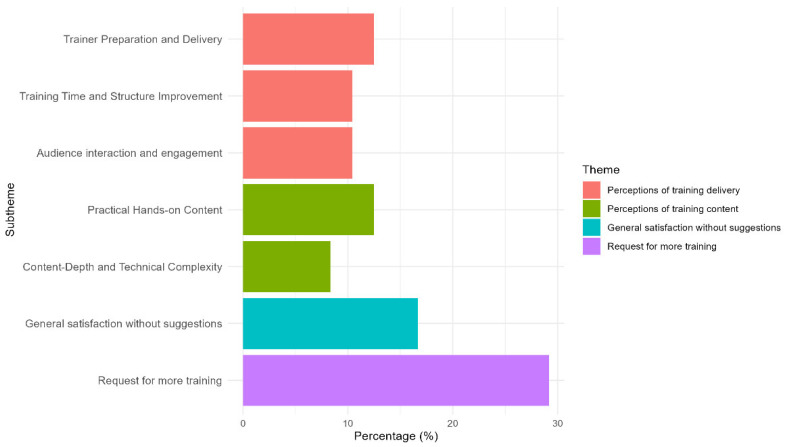
Distribution of Themes and Subthemes Derived from Livestock Workers’ Feedback on Training.

The qualitative analysis of post-survey responses revealed recurring themes related to training content, delivery, and personal learning outcomes. While most comments expressed appreciation for or satisfaction with the program, several livestock workers also shared constructive suggestions focused on improving topic depth, delivery clarity, and the inclusion of more hands-on activities. These findings provide valuable insight into participants’ experiences and help contextualize the quantitative knowledge gains observed across sectors. Figure 5 and Table 4 display the themes and subthemes derived from the livestock workers’ general suggestions and comments on the training.

### 3.10. Preferences for Future Training

To improve the design of future training sessions, farmworkers (*n* = 79) were asked about their preferences regarding the frequency, timing, length, format, and content. Table 5 shows that 30.4% prefer monthly training, 26.6% weekly, and 17.7% twice per month. Only 3.8% of participants felt that yearly training was enough. A total of 12.7% and 8.9% of the livestock workers expressed their willingness to receive training twice yearly and suggested doing the training every three months, respectively (Table 6).

Regarding the ideal time to receive the training, 40.5% preferred training after the workday, followed by 32.9% who preferred lunchtime sessions, and 26.6% favored early morning sessions.

When it comes to the duration of the training, 46.8% of the participants preferred 30-min sessions, followed by 27.8% who opted for 15-min sessions and 22.8% who would rather have 1-h sessions. Nearly all participants (98.78%) expressed a willingness and desire to continue receiving training.

Table 5 summarizes the livestock workers’ opinions about the training content and preferred formats for future education programs. It is important to note that livestock workers could choose multiple options for this question. When asked which topics were most useful, respondents most frequently mentioned acts of abuse (48.1%), biosecurity (46.8%), and humane euthanasia (44.3%). Other frequently mentioned topics included animal health (41.8%), animal handling (35.4%), and transport (29.1%).

Participants reported that the most appreciated aspects of the training were the use of images, graphics (20.3%), and the general layout of the presentations (16.5%). However, some areas for improvement were also noted, particularly the clarity of the information (10.1%). Preferred formats for future training include live demonstrations (19.0%) and instructional videos (16.5%). Topics suggested for future sessions included farmworker stress management (26.6%), farm technology use (24.1%), and effective communication (17.7%).

Most of the farmworkers (81.01%, *n* = 64) preferred receiving the presentations in Spanish. On the other hand, 12.65% (*n* = 10) of them preferred to receive the presentations in English. Furthermore, 6.32% (*n* = 5) are satisfied with receiving talks in both languages.

### 3.11. Interns’ Performance Across Livestock Sectors

Throughout the six-week training period, students underwent weekly evaluations. Participating students were evaluated based on eight instructional criteria and four overall performance indicators, as outlined in the methodology, to assess the impact of the training sessions across various livestock sectors.

Swine Sector: Evaluations from this sector show moderate variation in students’ performance. Some students demonstrated strong confidence and effective time management but received lower ratings for topic knowledge and task disposition. One student maintained a “good” rating, demonstrating confidence and strong Spanish communication skills. While areas for improvement were noted, both farm managers from the two farms expressed satisfaction with the students’ contributions.

Poultry Sector: Initial assessments revealed challenges in pronunciation, fluency, and engaging the audience. However, consistent improvement led to final evaluations that rated the students as “good” in all instructional areas. The farm manager rated overall performance as “excellent.”

Beef Cattle Sector: This sector exhibited a broader range of evaluation scores, with some students demonstrating exceptional preparedness while others faced significant challenges in audience interaction, fluency, and time management. Nevertheless, students excelled in confidence and Spanish communication. Farm managers rated overall performance as “good” and remarked that the students were eager to learn and motivated to communicate with farmworkers.

Dairy Sector: Students in the dairy sector consistently achieved high scores across many evaluation criteria. They were acknowledged for their strong knowledge of the topic, language abilities, confidence, and professionalism. Although there were occasional pronunciation and audience engagement difficulties, these did not affect their overall performance. Farm managers rated the students as excellent in punctuality, task disposition, and Spanish-language interaction. This sector was distinguished by its consistently high evaluations and positive learning atmosphere.

**Figure 6:**
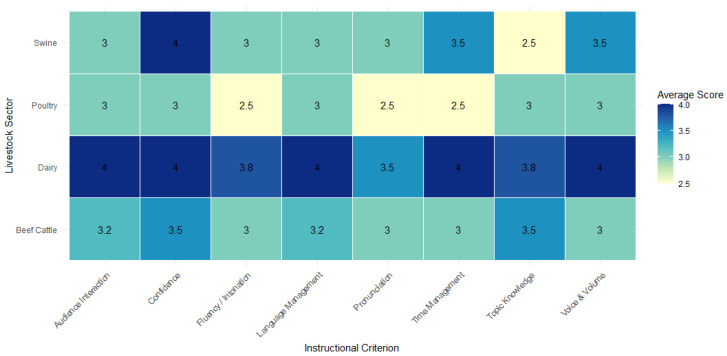
Livestock Workers’ Evaluation of Student Performance by Livestock Sector and Criteria.

Based on evaluation criteria, Figure 6 presents the average instructional performance scores across livestock sectors. The dairy sector demonstrated the highest consistency across all dimensions, with average scores ranging from 3.8 to 4.0, particularly reported as excellent in language use, confidence, and topic knowledge. In contrast, the poultry and swine sectors exhibited more variability, with students assigned to poultry farms scoring lower in fluency, interaction, and time management (averaging 2.5). Students in the swine sector showed stronger performance in confidence and voice volume but received lower ratings in topic depth (2.5). These patterns align with the qualitative evaluations from managers and reinforce sector-specific strengths and challenges in student-led delivery. Across all sectors, farm managers expressed their willingness to host more students, suggesting the overall success of the training model. Dairy showed the strongest performance consistency, while beef and swine presented more variation in specific areas like pronunciation, fluency, and time management. Language engagement was a clear strength across sectors, with all students rated as excellent or very good in their willingness to speak Spanish with workers.

## 4. Discussion

This study evaluated the impact of SSPA courses on Spanish-speaking livestock workers’ knowledge regarding animal welfare. The study explored how veterinary and animal science students, after completing such courses, delivered animal welfare presentations in Spanish to Spanish-speaking livestock workers across four different livestock sectors. The findings reveal that targeted language-based education in Spanish for these students can lead to measurable improvements in farmworkers’ knowledge, especially among less formally educated and less experienced farmworkers. The students’ on-farm internship also provided students with useful hands-on experience on daily farm operations, animal husbandry practices, biosecurity protocols, and valuable opportunities to put into practice and expand their acquired competence in Spanish through their daily interactions with Spanish-speaking livestock workers. This approach can contribute to strengthening insights for future training development and workforce support.

Several factors led to a decline in participants between the pre- and post-survey stages:

- Two swine farms (located in North Carolina) were not able to complete the post survey due to a disease outbreak that limited access to external personnel.
- One dairy farm withdrew from the project, stating that the assigned student did not meet their expectations. This situation underscores the importance of clear communication and shared understanding of goals during experiential learning placements.
- Additionally, two more farms opted not to conduct the post-surveys, although their participants had completed the training and pre-surveys. This was due to internal scheduling conflicts. The ethical protocol regarding voluntary participation respected this choice.

Overall, the training sessions, which were a component of the SSPA internship, effectively enhanced livestock workers’ knowledge, with notable improvements in key welfare areas, including acts of abuse and transport. The shift in average scores from 86% prior to training to 94% afterward highlights significant knowledge reinforcement, particularly among sectors and educational groups with initially lower understanding. Following a sector-specific review, dairy and poultry workers demonstrated the most significant knowledge improvements, at 8.0% and 7.4%, respectively, supporting previous studies that emphasize the effectiveness of practical, role-specific training for non-specialized employees [54,55]. However, beef cattle and swine workers showed limited advancements, due to a presumable “ceiling effect” caused by their high pre-existing knowledge, mainly by the groups represented by high education. This effect occurs when the subjects of a study have scores near the possible upper limit, preventing measurement or estimation of variance above a certain level [56]. These results highlight the need for assessing baseline knowledge before training delivery and adjusting content complexity accordingly. They also confirm the importance of designing sector-specific and role-adapted training content. Tailoring educational materials to reflect the baseline knowledge and daily responsibilities of each group may enhance learning outcomes and lead to more effective adoption of animal welfare practices on farms [13].

The strongest knowledge improvements were observed among participants with elementary education, confirming that when instructional content is context-specific and relevant, it can be effective even for learners with limited formal education [57]. These results support findings that visual aids, demonstrations, and culturally sensitive communication methods enhance understanding among adult learners with limited formal education or marginalized workforce groups [58,59,60].

In the analysis of knowledge assessment results across animal welfare topics, transport and animal health were identified as the areas with the highest proportion of individual improvement among livestock workers (21.52% and 15.19%, respectively). However, these topics also showed the highest percentages of worsened post-training scores (6.33% each). This apparent contradiction may be explained by the Dunning-Kruger effect, a cognitive bias in which individuals with low knowledge or expertise overestimate their understanding [61]. Before the training, some livestock workers may have responded with high confidence in the pre-survey despite having limited knowledge. After receiving the training, increased exposure to technical content may have led to greater self-awareness of knowledge gaps, resulting in more cautious responses in the post-survey. This shift reflects metacognitive calibration, where participants better recognize the complexity of a topic and become more critical of their knowledge, even if their accurate understanding has improved [62]. Therefore, the decrease in scores or worsening, as represented in Table 3, should not be interpreted strictly as a loss of knowledge but as part of a cognitive learning curve where learners reassess and refine their understanding.

Unexpected results also emerged in specific subgroups. One was the minimal or slightly negative improvement in knowledge scores among swine livestock workers. This outcome may be attributed not only to the strict biosecurity protocols [63], established welfare standards [46], and routine employee training common in swine operations, but also to the professional background of the workforce. A significant portion of these livestock workers are TN visa holders, many of whom are already trained in their country of origin as veterinarians or professionals in animal production. Their advanced baseline knowledge likely limited the measurable impact of the intervention. Although initially surprising, this finding reiterates the importance of knowledge pre-assessment before delivering training content, particularly for sectors with well-established welfare protocols. Tailoring educational programs to account for existing expertise could help avoid redundancy and promote engagement by introducing advanced or sector-specific modules for more experienced audiences.

Another unanticipated observation was the minor knowledge decline among participants with technical degrees, especially regarding transport stress management and animal handling. This finding could reflect several factors, including possible overconfidence in prior knowledge, differences between academic learning and practical farm practices, or even survey fatigue given the repetitive nature of pre- and post-assessments. Although the overall magnitude of decline was small, it suggests that future training programs should explicitly validate existing knowledge while encouraging continuous learning, even among participants with technical backgrounds. Reinforcing practical applications rather than theoretical content might better engage this demographic and bridge the gap between formal education and day-to-day farm operations.

Despite these variations in knowledge gain, multivariable logistic regressions indicated no statistically significant predictors of perceived training difficulty or satisfaction based on age, education, experience, or immigration status. This finding was surprising, as earlier research frequently indicated that educational background or language proficiency affects training receptivity. This contrasts with the results of Descovich et al. [64], where an analysis of the effects of animal welfare training on stakeholders’ knowledge in China revealed a significant difference in a multivariable analysis, indicating an effect of education level (*p* = 0.005) and years of experience (*p* < 0.0001).

One potential explanation for this outcome is the relatively small and homogenous sample size, which might have limited statistical power. Alternatively, it could be that providing Spanish training has created a more equitable environment, allowing farmworkers from various backgrounds to participate more equally in the content. If this is the case, the outcome reinforces the significance of culturally and linguistically tailored training programs to improve inclusion in workforce development.

Qualitative analysis of open-ended feedback revealed a strong appreciation for the practical application of the training. Workers valued the opportunity to enhance their understanding of animal care. While most feedback was positive, some participants underscored the significance of trainer preparedness and the depth of technical content. While most livestock workers expressed satisfaction with the training, a group preferred professional trainers, citing trainer preparedness and content depth as critical factors. This reflects a standard expectation in adult education that trainers possess subject-matter expertise and demonstrate strong presentation and engagement skills [65,66].

Manager evaluations of student presentations and performance indicated general satisfaction and a willingness across sectors to host future students. The dairy sector reported the most consistent and positive evaluations of its students. In contrast, some variability was noted in the swine and beef cattle sectors, primarily related to language fluency and time management. These insights suggest that enhancing Spanish-language proficiency in veterinary and animal science programs represents a viable and culturally appropriate approach to improving animal welfare knowledge among Hispanic livestock workers. However, the performance of each student may vary. The results of the students’ instruction performance highlight the feasibility and potential impact of integrating bilingual veterinary students into training programs for Spanish-speaking workers. They also identify key areas to be reinforced during student preparation, especially in communication strategies and classroom management under real farm conditions.

Results indicate that the workforce in this study primarily consists of less-experienced livestock workers, which may have implications for their training needs and the effectiveness of interventions. A significant portion of the sample (44.3%) has between 1 and 4 years of experience, suggesting that a young workforce could benefit from targeted training programs to improve their skills and understanding of animal welfare practices. The presence of a smaller proportion of highly experienced workers (20.2%) might imply the value of leveraging their expertise or advanced training activities.

The strong demand for more practical, hands-on sessions reflected in worker comments supports the growing need for experiential learning as a key methodology in farmworker education. Practical content allows immediate application, improving both retention and motivation. Gordon et al. [55] and Hernández Romero [54] reported similar preferences for hands-on and visual methods. Also, Central Valley- California livestock workers valued learning by doing, peer-to-peer mentoring, and short, recurring instructional moments. These informal daily learning methods help workers build relevant skills efficiently. In addition, a clinical study by Nguyen et al. [67] showed that Spanish-speaking farmworkers performed better in visual-spatial and short-term memory tasks, supporting the use of more visual and practical materials in future training.

The preference for live demonstrations and real-world examples indicates that traditional lecture-based methods may be less effective in this setting than more active and participatory approaches. This aligns with findings from Roman-Muniz et al. [13], which discussed issues in Colorado’s dairy operations. Their study involved informal surveys and observational feedback from producers to identify effective training strategies for livestock workers, showing that both didactic and hands-on laboratory experiences could prove beneficial. These results reinforce the importance of using experiential learning models instead of just theoretical or lecture-based formats in animal welfare education for adult livestock workers. These results are similar to those found in the Georgia Young Farmer Program and the adult education component of Georgia Agricultural Education, where the participants preferred kinesthetic learning (hands-on activities and practical demonstrations) [68]. Future training initiatives should prioritize interactive methods, including role-playing scenarios, problem-solving exercises, and case study discussions, to foster deeper engagement and enhance knowledge transfer to real-world farm environments.

Training programs must be rooted in adult learning theory. Andragogy outlines a framework of assumptions regarding how adults learn. This theory promotes problem-based and collaborative learning approaches rather than unidirectional content delivery (from trainer to learner). It emphasizes equality between teachers and learners, acknowledging that adults are internally motivated and self-directed and bring their life experiences and knowledge into the learning process [69]. They are also purposeful, value relevance, prefer practical applications, and seek respect as learners. Given the diverse educational backgrounds of the livestock workers involved in our project, we understand that a one-size-fits-all approach is inappropriate due to their varying educational levels. These differences can significantly affect satisfaction levels, perceptions of difficulty, and knowledge improvement. The training content was developed for livestock workers with a high school level of education, minimizing the inclusion of excessive scientific and technical details to avoid confusion, while ensuring that those with higher educational backgrounds, such as university or technical degrees, do not feel condescended to, however we didn’t expected to have high education farmworkers.

If the intern/trainer only has a theoretical knowledge base, this could affect their perceived credibility among participants, especially in professional or technical settings. Source credibility is typically evaluated through two dimensions: expertise (the communicator’s knowledge) and trustworthiness (the audience’s perception of their integrity and ability to apply that knowledge) [70]. In this program, the veterinary and animal science students served as trainers. While they may bring the theoretical content and enthusiasm to share their newly acquired knowledge, their limited field experience and developing language proficiency may influence how their credibility is perceived by the workers, particularly those with professional backgrounds, such as the TN visa holders. Research in science communication emphasizes that audiences value not only factual accuracy but also the communicator’s ability to contextualize information and relate it to real-world practice [71,72]. This gap between trainer experience and audience expertise may lead to reduced engagement or perceived relevance.

Another important consideration is that interns were not specifically prepared for professional-level presentations. While they were equipped with theoretical content and Spanish communicative skills, most did not specialize in animal welfare and were not trained to anticipate or respond to technical questions from professional participants. This gap in preparation may have affected their confidence and the perceived relevance of their delivery. Future training efforts could benefit from more targeted preparation that includes audience analysis, practice with potential questions, and deeper exposure to the subject matter.

The results demonstrate that livestock workers appreciated the initiative of student intervention training but identified clear areas for improvement. While the feedback was predominantly positive in learning and suggestions, critical feedback focused on better trainer preparation, deeper technical content, and more practical teaching strategies. The repeated call for “more practice” and “topic depth” suggests that future program versions should incorporate applied learning elements and tailored technical modules. The delivery of the training in the farmworkers’ native language may have contributed to the high levels of satisfaction and perceived usefulness, as reflected in qualitative feedback from most comments. The comparison of the two questions offers a comprehensive understanding of how participants evaluated the delivery, impact, and future training expectations, providing valuable guidance for future initiatives. The success of the student intervention training model in this study, positive manager evaluations, and willingness to host future students demonstrate that early-career professionals can be effective educators when appropriately prepared. This approach offers a scalable and cost-effective strategy for expanding welfare education across the industry while providing valuable field experience for students.

While this study focused on training livestock workers, future efforts to educate students and consumers about animal welfare could benefit from incorporating digital platforms. For example, social media tools like Instagram have been used effectively to communicate complex agricultural topics to broader audiences, supporting science communication and public engagement alongside formal instruction [73]

Delivering training in Spanish was critical in ensuring accessibility and comprehension among the Spanish-speaking livestock worker population. The minimal impact of demographic variables on perceived training difficulty and satisfaction suggests that offering content in workers’ native language significantly levels the educational playing field. Agricultural training initiatives should prioritize language accessibility in delivery and materials design, evaluation tools, and follow-up support. Moreover, recognizing the different learning preferences between male and female workers can help refine instructional methods to be even more inclusive and effective.

Despite these valuable insights, several limitations should be acknowledged. First, not all farms that initially agreed to participate completed the pre- and post-survey phases, reducing the final sample size and introducing selection bias. Furthermore, the farm manager’s role in choosing participants might have biased the findings. Only skilled or high-achieving workers were selected at some farms, like beef cattle, leading to inflated baseline scores and a diminished observable knowledge increase. Conversely, other farms permitted participation from all workers, presenting a more accurate representation of the training effects across various experience levels. There was also a notable variation in worker literacy and comprehension levels. Although the surveys were read aloud in Spanish to cater to diverse literacy levels, some workers needed additional clarification. This indicates that upcoming surveys might benefit from considering alternative formats, like visuals or verbal quizzes, to reduce the cognitive load on participants with lower reading comprehension. Finally, this study assessed knowledge after the intervention but lacked long-term follow-up. Although short-term improvements were noted, it is still unclear if workers kept the knowledge over time or if it led to behavioral changes or better animal outcomes. Follow-up research is required to evaluate retention and lasting effects [13].

## 5. Conclusions

This study demonstrates that the SSPA curriculum effectively enhanced veterinary and animal science students’ ability to deliver impactful animal welfare training to Spanish-speaking livestock workers. After completing three SSPA courses, students successfully conducted animal welfare training sessions on swine, poultry, beef and dairy cattle farms, resulting in measurable self-perceived knowledge gains—especially among workers with limited formal education—on critical topics such as abuse prevention and transport practices.

Knowledge improvement varied by species, with dairy cattle and poultry workers achieving the highest gains, while beef and swine workers showed a “ceiling effect” due to prior training. Beyond improving farmworkers’ knowledge, the program provided students with valuable hands-on experience, deepening their understanding of animal husbandry, biosecurity, and cross-cultural communication.

These findings underscore the potential of integrating language-based education into animal science and veterinary curricula as a scalable, low-cost approach to bridge communication gaps, improve animal welfare, and strengthen workforce development in livestock production. The results suggest that partnerships between universities and farms can create mutually beneficial learning opportunities, fostering both student professional growth and more inclusive, effective on-farm training. This study also suggests several implications for the design, delivery, and refinement of farmworker training programs based on previously identified needs, particularly in linguistically and culturally diverse livestock environments.

In sum, this study highlights a promising viable model for preparing prospective animal science and veterinary professionals to address the linguistic and cultural needs that arise in the framework of diverse livestock operations—ultimately driving progress in animal care and welfare.

## Figures and Tables

**Table 1:** Sociodemographic characteristics of farmworkers by livestock sector (*n* = 79).

Categories		Livestock Sectors			
		Swine (*n* = 18)	Poultry-Laying Hens (*n* = 22)	Beef Cattle (*n* = 12)	Dairy Cattle (*n* = 27)
Gender	Female	12 (66.67%)	12 (54.55%)	0	10 (37.03%)
	Male	6 (33.33%)	10 (45.45%)	12 (100%)	17 (62.97%)
Education	Elementary School	1 (5.55%)	11 (50%)	0	8 (29.62%)
	High School	5 (27.77%)	9 (40.90%)	1 (8.34%)	8 (29.62%)
	Technical Degree	0	0	0	4 (14.82%)
	University Degree	12 (66.66%)	2 (9.10%)	11 (91.66%)	7 (25.94%)
Nationality	Mexico	16 (88.89%)	16 (72.71%)	11 (91.67%)	23 (85.18%)
	Guatemala	0	2 (9.09%)	0	4 (14.82%)
	USA	2 (11.11%)	4 (18.20%)	1 (8.33%)	0
Bilingual	Yes	7 (38.88%)	14 (63.63%)	4 (33.33%)	1 (3.70%)
	No	11 (61.11%)	8 (36.37%)	8 (66.67%)	26 (96.30%)
Age	18–35	11 (61.11%)	7 (31.81%)	10 (83.34%)	23 (85.19%)
	36–55	6 (33.33%)	9 (40.90%)	2 (16.66%)	4 (14.81%)
	>56	1 (5.56%)	6 (27.29%)	0	0
Migration Status	Visa TN	15 (83.33%)	1 (4.56%)	11 (91.66%)	11 (40.74%)
	Permanent Resident-Citizen	2 (11.11%)	14 (63.63%)	0	2 (7.40%)
	I would rather not answer	1 (5.55%)	7 (31.81%)	1 (8.34%)	14 (51.85%)

**Table 2:** Livestock Workers’ Knowledge Gain by Animal Welfare Topic.

Topic	Questions	Pre (%)	Post (%)	Knowledge Gain (%)
Euthanasia	Question 1	68.35	77.22	8.86
	Question 2	89.87	89.87	0.00
	Question 3	86.08	88.61	2.53
Acts of Abuse	Question 4	84.81	97.47	12.66
	Question 5	89.87	96.20	6.33
Animal Handling	Question 6	82.28	88.61	6.33
	Question 7	96.20	98.73	2.53
Biosecurity	Question 8	87.34	97.47	10.13
	Question 9	91.14	97.47	6.33
Animal Health	Question 10	72.15	82.28	10.13
	Question 11	88.61	94.94	6.33
Records	Question 12	94.94	96.20	1.27
	Question 13	82.28	87.34	5.06
Feeding and Water Intake	Question 14	94.94	94.94	0.00
	Question 15	88.6	92.4	3.80
Lameness	Question 16	98.7	100.0	1.27
	Question 17	93.67	100.00	6.33
Transport	Question 18	65.82	79.75	13.92
	Question 19	77.22	91.14	13.92
Housing	Question 20	94.94	97.47	2.53
	Question 21	98.73	97.47	−1.27
Mortality	Question 22	94.94	97.47	2.53
	Question 23	97.47	98.73	1.27
Behavior	Question 24	96.20	97.47	1.27
	Question 25	88.61	93.67	5.06

**Table 3:** Themes and Subthemes Derived from Livestock Workers’ Perceived Knowledge Gain.

Theme	Subthemes	Description	Quotes	*n*	Frequency	Individual Percentage	Percentage	Intercoder Reliability
Acquisition of new knowledge	Learning new content	Farmworkers acknowledged that they had gained knowledge on topics they had not encountered before	“*We learned some stuff I did not know before.*” (P7).	29	23	50	63	0.831
	Clarification of previous doubts	Farmworkers highlighted that the training helped them understand concepts they previously found confusing.	“*I used to have doubts before, now I understand*” (P59)		6	13.04		
Training expectations and evaluation	Specific Content required	Participants expressed the need for specific topics to be expanded, particularly regarding the animal behavior of their specific species	“*Learning a little bit more about animal behavior*” (P9)	10	4	8.7	21.8	
	Preferences for training	Participants preferred trainers with whom they could personally relate, such as those of a similar age or background, which enhanced their learning experience.	“*I learned better with someone my age,*” (P64)		2	4.35		
	More practical content	Feedback indicated a preference for more hands-on learning opportunities and demonstrations during the training.	“*Incorporate more practical exercises into the training,*” (P55)		4	8.7		
Feedback from past knowledge		Participants felt the sessions helped them reinforce previously learned concepts from formal education or experience.	“*I revisited topics from college/university.*” (P12)	7			15.2	
				N total	46			

**Table 4:** Themes and Subthemes Derived from Livestock Workers’ General Suggestions and Comments about the Training.

Theme	Sub-Theme	Description	Quotes	*n*	Frequency	Individual Percentage	Percentage	Intercoder Reliability
Perceptions of training delivery.	Trainer Preparation and Delivery	This captures the farmworkers’ observation about the student’s preparedness, professionalism, and delivery style.	“*They should prepare before presenting. The topics were good, but they did not know how to explain them*.” P26.	16	6	12.5	33.33%	0.887
	Training Time and Structure Improvement	This one reflects suggestions related to scheduling, length, and organization of the training sessions.	“*Set a specific schedule for the training and cover all the important points in farm management,*” P29		5	10.42		
	Audience interaction and engagement	Feedback on how engaged farmworkers felt during the sessions and the level of interaction encouraged by the students as trainers	“*Students should engage more actively by asking questions to the audience and be less nervous*,” *P42*		5	10.42		
Request for more training		Farmworkers desire to receive additional training opportunities in the future.	“*It was good, and it would be even better with a follow-up to improve animal handling*,” *P56*	14	14	29.17%		
Perceptions of training content	Practical Hands-on Content	Feedback on the applicability of training materials. Required more practice-based explanations.	“*More educational materials and more live demonstrations*,” P10	10	6	12.5	20.83%	
	Content-Depth and Technical Complexity	Farmworkers’ reflections on how understandable the training content was, deeper training is required	“*Approach the training with more technical or comprehensive situations,*” P46.		4	8.33		
General satisfaction without suggestions		Farmworkers were satisfied with the training and did not provide further suggestions.	“*Everything was excellent*,” P8	8	8	16.67%		
				N total	48			

**Table 5:** Livestock Workers’ Preferences for Future Training.

Category	Response	Frequency	*n*	Percentage	(95% CI)
Preferred Training Frequency	Once per year	3	79	3.8	1.3–10.8
	Twice per year	7		8.9	4.5–17.6
	Every three months	10		12.7	7.2–22.3
	Once per month	24		30.4	19.7–39.5
	Twice per month	14		17.7	11.2–28.2
	Once per week	21		26.6	18.6–38.1
Preferred Time of Day	Before work	21	79	26.6	18.6–38.1
	During lunch	26		32.9	21.9–42.2
	After work	32		40.5	31.2–52.7
Preferred Training Duration	15 min	22	79	27.8	19.7–39.5
	30 min	39		49.4	37.3–59.0
	1 h	18		22.8	15.3–34.0

**Table 6:** Livestock Workers’ Evaluation of Training Content and Preferred Formats for Future Education Programs.

**Category**	**Response**	**Frequency**	**Percentage**	**(95% CI)**
Topics Learned the Most	Acts of abuse	38	48.1	37.4–58.9
	Biosecurity	37	46.8	36.2–57.7
	Animal health	33	41.8	31.5–52.8
	Humane euthanasia	35	44.3	33.9–55.3
	Animal handling	28	35.4	25.8–46.4
	Transport	29	36.7	26.9–47.7
	Lameness	20	25.3	17–35.9
	Feeding & water	20	25.3	17–35.9
	Behavior	20	25.3	17–35.9
	Mortality	12	15.2	8.9–24.7
	Housing	12	15.2	8.9–24.7
	Records	10	12.7	7–21.8
Liked Aspects of the PPT	Use of images/graphics	16	20.3	12.9–30.4
	Visual design	13	16.5	9.9–26.1
	Clarity of information	11	13.9	8–23.2
	Content organization	6	7.6	3.5–15.6
Aspects to Improve the PPT	Visual design	9	11.4	6.1–20.3
	Clarity of information	8	10.1	5.2–18.7
	Use of images/graphics	6	7.6	3.5–15.6
	Content organization	3	3.8	1.3–10.6
Preferred Training Formats	Live demonstrations	15	19.0	11.9–29
	Instructional videos	13	16.5	9.9–26.1
	Written guides	8	10.1	5.2–18.7
	Interactive apps	5	6.3	2.7–14
Suggested Future Topics	Farmworker Stress Management	21	26.6	18.1–37.2
	Farm technology use	19	24.1	16–34.5
	Effective communication	14	17.7	10.9–27.6
	Sustainability/environment	14	17.7	10.9–27.6
	Workplace safety	13	16.5	9.9–26.1

## Data Availability

All data included in the manuscript are available at Garcia’s Laboratory.

## Appendix A

Question A1. Pre and Post Evaluation Survey for Livestock Workers: Demographic questions (English Version)

Please select or write the information that applies to your situation:

1. Age: ________________________

2. Country of origin: _________________________

3. Sex: ___ Male ___ Female ___ Other

4. Education:

____ Completed elementary school ____ Completed high school

____ Technical degree ____ University degree

Other: ________________________

5. Have you received training in animal handling?

____ Yes ____ No

6. Position on your current farm: ________________________________

Years in current position: ____

7. How long have you been working at this company? ____ years _____ months

8. Do you have previous experience working with animals?

Yes_______ No ___________

Type of work: __________________________________________________

Place: _________________________________ Duration: _______________

9. Animal species you work with:

____ Dairy cattle ____ Beef cattle

____ Broiler chickens ____ Pigs

____ Laying hens

Other: _______________________________

10. Languages you speak:

____ English ____ Spanish Others: ___________________________________

## Appendix B

The purpose of this survey is to identify your knowledge about animal handling, animal welfare, and food safety. Your responses will be used solely for statistical and research purposes and are completely anonymous. Your participation is voluntary. There will be no consequences for responding to this survey. Your opinion is valuable. Thank you very much for your time. Please answer Yes or No.

**Table A1:** Pre and Post Evaluation Survey for Livestock Workers: Knowledge Assessment Animal Handling, Welfare, and Food Safety.

		Yes	No
1	I am responsible for the euthanasia process on the farm, and I know the procedure to carry it out.		
2	I know when an animal is sick or injured and requires euthanasia.		
3	I know how to verify that euthanasia has been performed correctly and that the animal is dead.		
4	I know what constitutes an act of animal abuse.		
5	I understand what animal neglect means.		
6	I understand what the flight zone is.		
7	I know that if I move the animals at a calm and steady pace (without yelling or hitting), they will experience less stress.		
8	I understand that when an animal arrives at the farm, it is important to separate it from the rest for a period of time to prevent the spread of any disease.		
9	I know what the biosecurity terms ‘clean zone’ and ‘dirty zone’ on a farm mean.		
10	I understand what the withdrawal period of certain medications consists of.		
11	I know that if I do not follow the veterinarian’s instructions regarding the use of antibiotics in animals, I can negatively affect human health.		
12	I understand that records are important to help identify, treat, and prevent disease outbreaks.		
13	I know that records are used to track the use of antibiotics and the withdrawal times of medications when the meat of the animals will be consumed by humans.		
14	I understand that water and feed intake is based on the animal’s health status. (For example: if an animal is sick, it may not eat or drink as much as when it is healthy).		
15	I know how to identify when an animal has a low body condition.		
16	I know how to identify an animal that has a leg injury.		
17	I understand that lameness or leg injuries can compromise the health of animals and lead to death.		
18	I understand that actions that I need to perform in order to prevent heat exposure or cold stress.		
19	I know which animals are fit for transport according to their health status.		
20	I know it is important to report when equipment is not working properly, as its use can harm the health or life of the animals (ventilation systems, etc.).		
21	I understand that it is my responsibility to identify and report any maintenance failures in the facilities that could result in injuries or harm to the animal.		
22	I know that dead animals must be disposed of according to the farm’s standardized operating procedures.		
23	I understand that the animal’s death must be verified before taking it to the disposal area.		
24	I know how to identify abnormal behaviors that indicate the animal is sick or stressed.		
25	I know how to identify injuries caused by abnormal behaviors (e.g., if they are the result of pecking in birds, tail biting in pigs, etc.).		

## Appendix C

**Table A2:** Pre and Post Evaluation Survey for Livestock Workers: Animal Welfare Knowledge Assessment, questions organized by Topics.

Topic	Questions	
**Euthanasia**	Question 1	I am responsible for the euthanasia process on the farm, and I know the procedure to carry it out.
	Question 2	I know when an animal is sick or injured and requires euthanasia.
	Question 3	I know how to verify that euthanasia has been performed correctly and that the animal is dead.
**Acts of Abuse**	Question 4	I know what constitutes an act of animal abuse.
	Question 5	I understand what animal neglect means.
**Animal Handling**	Question 6	I understand what the flight zone is.
	Question 7	I know that if I move the animals at a calm and steady pace (without yelling or hitting), they will experience less stress.
**Biosecurity**	Question 8	I understand that when an animal arrives at the farm, it is important to separate it from the rest for a period of time to prevent the spread of any disease.
	Question 9	I know what the biosecurity terms ‘clean zone’ and ‘dirty zone’ on a farm mean.
**Animal Health**	Question 10	I understand what the withdrawal period of certain medications consists of.
	Question 11	I know that if I do not follow the veterinarian’s instructions regarding the use of antibiotics in animals, I can negatively affect human health.
**Records**	Question 12	I understand that records are important to help identify, treat, and prevent disease outbreaks.
	Question 13	I know that records are used to track the use of antibiotics and the withdrawal times of medications when the meat of the animals will be consumed by humans.
**Feeding and Water Intake**	Question 14	I understand that water and feed intake is based on the animal’s health status. (For example: if an animal is sick, it may not eat or drink as much as when it is healthy).
	Question 15	I know how to identify when an animal has a low body condition.
**Lameness**	Question 16	I know how to identify an animal that has a leg injury.
	Question 17	I understand that lameness or leg injuries can compromise the health of animals and lead to death.
**Transport**	Question 18	I understand that actions that I need to perform in order to prevent heat exposure or cold stress.
	Question 19	I know which animals are fit for transport according to their health status.
**Housing**	Question 20	I know it is important to report when equipment is not working properly, as its use can harm the health or life of the animals (ventilation systems, etc.).
	Question 21	I understand that it is my responsibility to identify and report any maintenance failures in the facilities that could result in injuries or harm to the animal.
**Mortality**	Question 22	I know that dead animals must be disposed of according to the farm’s standardized operating procedures.
	Question 23	I understand that the animal’s death must be verified before taking it to the disposal area.
**Behavior**	Question 24	I know how to identify abnormal behaviors that indicate the animal is sick or stressed.
	Question 25	I know how to identify injuries caused by abnormal behaviors (e.g., if they are the result of pecking in birds, tail biting in pigs, etc.).

## Appendix D

Question A2. Post Intervention Evaluation Survey for Livestock Workers: Training Feedback and Preferences (English Version)

General Questions

We appreciate your willingness to participate in the trainings provided by the students. We would like to ask you a few questions about how the sessions were conducted.

1. Perceived level of difficulty of the trainings

(a) Not difficult

(b) Slightly difficult

(c) Moderately difficult

(d) Very difficult

(e) Extremely difficult

2. On a scale from 1 to 5, how satisfied are you with receiving the training from students?

(a) Not satisfied

(b) Slightly satisfied

(c) Satisfied

(d) Very satisfied

(e) Extremely satisfied

3. Would you have preferred to receive the training from a professional?

YES___________ NO __________

If yes, please explain why: ____________________________________

4. Do you feel that your knowledge has improved as a result of the trainings?

YES___________ NO____

Why? ________________________________________________________

5. Please write any suggestions or comments to improve the trainings:

____________________________________________________________

6. After the sessions, did you notice any difference in your daily work?

For example: Becoming more aware at work of a topic you didn’t understand before.

YES ________________________

NO ________________________

7. How often would you like to receive trainings?

(a) Once a week

(b) Twice a month

(c) Once a month

(d) Once every 3 months

(e) Twice a year

(f) Once a year

8. What time of day do you prefer to receive training?

(a) In the morning before work

(b) At midday during lunch

(c) After the workday ends

(d) Other: ____________________________

9. How long would you prefer each training session to last?

(a) 15 min

(b) 30 min

(c) 1 h

(d) Other: ____________________________

10. Would you be willing to receive more trainings in the future?

(a) Yes

(b) No

11. Select the topics in which you feel you learned something new (you may select more than one):

(a) Acts of abuse

(b) Biosecurity

(c) Animal health

(d) Lameness

(e) Water and feed

(f) Housing

(g) Humane euthanasia

(h) Recordkeeping

(i) Transportation

(j) Behavior

(k) Animal handling

(l) Recordkeeping

12. Do you prefer to receive this type of training in:

(a) English

(b) Spanish

13. Did you find the PowerPoint content useful?

(a) Not useful

(b) Slightly useful

(c) Useful

(d) Very useful

(e) Extremely useful

14. What aspects of the PowerPoint presentations did you like the most? (You may select more than one):

(a) Visual design

(b) Clarity of information

(c) Use of images and graphics

(d) Organization of content

(e) Other: ______________________

15. What aspects of the PowerPoint presentations would you like to see improved? (You may select more than one):

(a) Visual design

(b) Clarity of information

(c) Use of images and graphics

(d) Organization of content

(e) Other: ______________________

16. Would you like to receive the training content in another format besides PowerPoint? (You may select more than one):

(a) Instructional videos

(b) Written manuals or guides

(c) Interactive apps

(d) Live sessions with demonstrations

(e) Other: ______________________

17. What other topics would you like to see included in future trainings? (You may select more than one):

(a) Workplace safety

(b) Stress management

(c) Effective communication

(d) Use of technology on the farm

(e) Sustainability and the environment

(f) Other: ______________________

18. What is your immigration status?

(a) Work visa (NAFTA or TN type)

(b) Temporary resident

(c) Permanent resident

(d) U.S. citizen

(e) Prefer not to answer

## Appendix E

**Table A3:** Topics for Swine Production.

#	Topics—Swine	Description
1	Euthanasia	What is euthanasia? euthanasia plans for each farm, which animals should get euthanasia? euthanasia methods for pigs, how to clean the euthanasia equipment properly, and how to confirm the insensibility and death of the animal?
2	Acts of Abuse	What is considered an act of abuse? Examples of acts of abuse include neglecting care for animals. What should I do if I witness an act of abuse? How to proceed with non-ambulatory animals.
3	Animal Handling	Specific characteristics of pigs: understanding pigs. Understanding the behavior of pigs, movement, tools for moving them, behavior of animal operators, preventive measures, and how to reduce hostile management. Stress indicators during management. Blind spot, flight zone.
4	Biosecurity	Fundamental aspects of biosecurity, the request for farms to have a biosecurity plan, what should be included in a biosecurity plan, internal biosafety, procedures for new people entering the farm, the concept of clean areas and dirty areas, culled Animals, plan for cleaning and disinfection, needle disposal, external biosecurity, potential sources of pathogens, high-risk practices on farms.
5	Animal Health	Procedures to deal with sick animals on farms, abscesses, open wounds, tail bites, sores on the shoulders, daily inspection routine, vulvar lesions, health plan for the herd, segregation, quarantine or isolation of sick animals, hospital pen, specific characteristics a sick pen should have.
6	Records	Daily records, treatment records, mortality records, vaccine records, farmworker training records, and inventory of medicines records.
7	Feeding and Water Intake	Feeding, cleaning the feeders, types of feed the animals cannot take, drinkers, nipples, how much water a pig needs daily, daily inspection routine on farms, body condition score in piglets, sows, and pigs.
8	Lameness	What is lameness, what are its causes, what are the clinical signs of lameness, what is the locomotion score, what is the treatment, and what is analgesic use?
9	Transport	Cleaning and disinfection of the transport, summer, and winter conditions require space to transport the animals, preparing the animals for moving day, and loading the pigs.
10	Housing	What are the basic necessary conditions for housing pigs, and how can they identify if there is a risk for the animal according to the housing conditions?
11	Mortality	Mortality records, what to do if I find a dead animal, procedures to move animals heavier than 45 pounds, when to remove mortality, safety elements necessary to handle mortality, routine mortality, catastrophic mortality, proper methods of mortality disposal, things to consider in the disposal of mortality, how to double-check a dead animal.
12	Behavior	Why it is important to understand the behavior of pigs, animal behavior, how to deal with aggressive sows, sows in farrowing crates, tail biting, stereotypies.

## Appendix F

**Table A4:** Topics for Laying Hens Production.

#	Topics—Laying Hens	Description
1	Euthanasia	Critical points in humane euthanasia, performing euthanasia on time, identifying which animals should undergo euthanasia, training personnel for euthanasia, handling euthanasia equipment, and ways to verify euthanasia, examples of animal candidates for euthanasia, and euthanasia records.
2	Acts of Abuse	What is considered an act of abuse? Examples of acts of abuse include neglecting care for animals. What should I do if I witness an act of abuse? How to proceed with non-ambulatory animals.
3	Animal Handling	Considerations for handling animals, characteristics about hens to consider, training of personnel, how to handle the birds with the legs, how to reduce the risk of bone fractures, proper handling to minimize stress.
4	Biosecurity	Biosecurity and sanitation plans. What are some ways to promote biosecurity? A cleaning and sanitation plan, a waste disposal plan, animal quarantine, a clean zone, and a dirty zone.
5	Animal Health	What is an animal health plan? Record keeping, animal monitoring, abnormal behaviors, and animal health plan.
6	Records	Daily records, treatment records, mortality records, vaccine records, farmworker training records, and inventory of medicines records.
7	Feeding and Water Intake	Feeding, cleaning the feeders, types of feed the animals cannot take, drinkers, and the daily farm inspection routine.
8	Lameness	What is lameness, what are its causes, what are the clinical signs of lameness, what is the locomotion score, what is the treatment, and what is analgesic use?
9	Transport	Capture and handling of birds, what to do on the farm to catch the birds, loading them, withdrawal of feed and supplements, special equipment for catching birds, type of cages, bird density, loading the truck, required climate conditions during transport, duration of the process, water/ feed, climate control measures, water withdrawal and feeding conditions, specific conditions of capture and handling, identification of animals unfit for transport.
10	Housing	Hen inspections, identify and record the number of deaths and culls/injured/sick birds with stated reasons, equipment inspections and maintenance, water system inspections, auxiliary power source inspections (generators), ventilation and environmental controls, ammonia level monitoring, litter maintenance plan.
11	Mortality	Mortality records, what to do if I find a dead animal, procedures to move animals, when to remove mortality, safety elements necessary to handle mortality, routine mortality, catastrophic mortality, proper methods of mortality disposal, things to consider in the disposal of mortality, how to double-check a dead animal.
12	Behavior	Understanding behavior and how to deal with aggressive animals, pecking in birds.

## Appendix G

**Table A5:** Topics for Beef Cattle Production.

#	Topics—Beef Cattle	Description
1	Euthanasia	Critical points in humane euthanasia, identifying which animals should undergo euthanasia, training personnel for euthanasia, handling euthanasia equipment, and ways to verify euthanasia
2	Acts of Abuse	What is considered an act of abuse? Examples of acts of abuse include neglecting care for animals. What should I do if I witness an act of abuse? How to proceed with non-ambulatory animals.
3	Animal Handling	Use of instruments to move cattle, understanding vocalizations during handling, handler behavior and demeanor, use of lighting in animal movement, environmental management (visual distractions and shadows), and animal movement technique, birth and weaning Care, colostrum, benefits of colostrum, causes that can reduce colostrum quality, birthing areas, thermal environment, other conditions during birth, care for birthing problems, assistance during birth, postpartum evaluation of the cow, weaning, monitoring of spaces during the birthing period, removal of supernumerary teats in cattle, dehorning, tipping of horns, castration.
4	Biosecurity	Fundamental aspects of biosecurity, the request for farms to have a biosecurity plan, what should be included in a biosecurity plan, internal biosafety, procedures for new people entering the farm, the concept of clean areas and dirty areas, culled animals, plan for cleaning and disinfection, needle disposal, pest and predator control, potential sources of pathogens, high-risk practices on farms.
5	Animal Health	A daily inspection routine: water, feed, air and ground conditions, daily monitoring, non-ambulatory animals, leg condition, tail condition, internal and external parasite control, bald spots in cattle, hygiene evaluation, herd health plan.
6	Records	Daily records, treatment records, mortality records, vaccine records, farmworker training records, and inventory of medicines records.
7	Feeding and Water Intake	Feeding, cleaning the feeders, types of feed the animals cannot take, drinkers, how much the cattle need daily, daily farm inspection routine, and body condition score.
8	Lameness	What is lameness, what are its causes, what are the clinical signs of lameness, what is the locomotion score, what is the treatment, and what is analgesic use?
9	Transport	Cleaning and disinfection of the transport, understanding cattle behavior during the transport (blind spot and flight zone), summer and winter conditions, required space to transport the animals, preparing the animals for moving day, and loading animals.
10	Housing	Description of housing conditions, inspection of housing facilities, care needs to be taken, rest and shade area, high temperatures, humidity, and manure management.
11	Mortality	Mortality records, what to do if I find a dead animal, procedures to move animals, when to remove mortality, safety elements necessary to handle mortality, routine mortality, catastrophic mortality, proper methods of mortality disposal, things to consider in the disposal of mortality, how to double-check a dead animal.
12	Behavior	Understanding cattle behavior and how to deal with aggressive animals, head butting pushing, mounting.

## Appendix H

**Table A6:** Topics for Dairy Cattle Production.

#	Topics—Dairy Cattle	Description
1	Euthanasia	Critical points in humane euthanasia, identifying which animals should undergo euthanasia, training personnel for euthanasia, handling euthanasia equipment, and ways to verify euthanasia
2	Acts of Abuse	What is considered an act of abuse? Examples of acts of abuse include neglecting care for animals. What should I do if I witness an act of abuse? Evaluation and how to proceed with non-ambulatory animals.
3	Animal Handling	Flight zone, blind spot, use of flags to move the animals, extra advice about moving the animals, eliminating visual distractions during movement, behavior of animal handler, understanding vocalizations of cows, management of newborn calves, colostrum, and its benefits, causes that reduce the quality of colostrum, disinfecting the umbilical cordon, water and space required for calves, evaluating body condition score in calves, adequate techniques for milking process.
4	Biosecurity	Biosecurity and sanitation plans. What are some ways to promote biosecurity? A cleaning and sanitation plan, a waste disposal plan, animal quarantine, a clean zone, a dirty zone, needle disposal, and animal vaccines.
5	Animal Health	What is an animal health plan? Record keeping, animal monitoring, abnormal behaviors, and animal health plan.
6	Records	Daily records, treatment records, mortality records, vaccine records, farmworker training records, and records of the inventory of medicines.
7	Feeding and Water Intake	Feeding, cleaning the feeders, types of feed the animals cannot take, drinkers, and the daily farm inspection routine.
8	Lameness	What is lameness, what are its causes, what are the clinical signs of lameness, what is the locomotion score, what is the treatment, and what is analgesic use?
9	Transport	Cleaning and disinfection of the transport, understanding cattle behavior during the transport (blind spot and flight zone), summer and winter conditions, required space to transport the animals, preparing the animals for moving day, and loading animals.
10	Housing	Thermal environment for calves, disinfection of their space and bed management.
11	Mortality	Mortality records, what to do if I find a dead animal, procedures to move animals, when to remove mortality, safety elements necessary to handle mortality, routine mortality, catastrophic mortality, proper methods of mortality disposal, things to consider in the disposal of mortality, how to double-check a dead animal.
12	Behavior	Field observation of behavior, understanding the visual space of the animals, and the position of the head of the animals that indicates specific behavior, shadows, and lights, recognizing signs of discomfort in cows, recognizing normal behavior in cows, excessive vocalization, overgrooming, stereotypies.

## References

[1] Pietrosemoli S., Tang C. (2020). Animal Welfare and Production Challenges Associated with Pasture Pig Systems: A Review. Agriculture.

[2] Arcury T.A., Estrada J.M., Quandt S.A. (2010). Overcoming language and literacy barriers in safety and health training of agricultural workers. J. Agromed..

[3] Borys A., Ingram P.D. Spanish for the Agricultural Industries: Lessons from an Immersion Experience. Proceedings of the 24th Annual Conference of the Association for International Agricultural and Extension Education (AIAEE).

[4] Schenker M., Gunderson P. (2013). Occupational Health in the Dairy Industry Needs to Focus on Immigrant Workers, the New Normal. J. Agromed..

[5] Carroll D., Samardick R.M., Bernard S., Gabbard S., Hernandez T. (2005). A demographic and employment profile of United States farm workers. Findings from the National Agricultural Workers Survey (NAWS) 2001–2002.

[6] Clift C. (2021). We Speak English Here: An Exploratory Study of Language Barrier Effects in Agriculture. Bachelor’s Thesis.

[7] Garcia-Pabon J. (2011). Managing Latino Labor in the Pork Industry. Ph.D. Thesis.

[8] Zong J., Batalova J. (2015). The Limited English Proficient Population in the United States.

[9] Viveros-Guzmán A., Gertler M. (2015). Latino Farmworkers in Saskatchewan: Language Barriers and Health and Safety. J. Agromed..

[10] Hemsworth P.H., Coleman G.J. (2010). Human-Livestock Interactions: The Stockperson and the Productivity of Intensively Farmed Animals.

[11] George A.J., Bolt S.L. (2023). The importance of the human–animal relationship for commercial farms. Livestock.

[12] Maréchal L., Barcelos A.M., Cole J., King M. (2024). Human–Animal Welfare: The Interconnectedness of Human Well-Being and Animal Welfare. Introduction to Human-Animal Interaction.

[13] Roman-Muniz I.N., Metre D.C.V., Hirst H.L., Garry F.B. (2004). Training Spanish-speaking livestock workers: Theory and practice. Bov. Pract..

[14] Vaarst M., Roderick S., Smolders G., Leeb C., Walkenhorst M., Winckler C., Gratzer E., Stöger E., Whistance L.K., Brinkmann J. (2011). The dialogue with farmers. The Process of Minimising Medicine Use Through Dialogue Based Animal Health and Welfare Planning: Workshop Report-Core Organic Project nr.: 1903-ANIPLAN.

[15] Honig E. (2018). With Spanish Classes, Vet Schools Aim to Break Down Barriers with Farmworkers.

[16] Hemsworth P.H. (1997). Human-animal interactions in agriculture and their impact on animal welfare and performance. BSAP Occas. Publ..

[17] Ennis S.R., Ríos-Vargas M., Albert N.G. (2011). The Hispanic Population: 2010.

[18] Fooks L. (2021). Danger In The Fields: How Hispanic Farm Workers Are Affected by Hazardous Workplace Chemicals Due to a Lack of English Proficiency. https://nchcureca.com/danger-in-the-fields-how-hispanic-farm-workers-are-affected-by-hazardous-workplace-chemicals-due-to-a-lack-of-english-proficiency/.

[19] Noe-Bustamante L., Lopez M.H., Krogstad J.M.U.S. (2020). Hispanic Population Growth Slows After Surpassing 60 Million in 2019.

[20] Ramos A.K., Adhikari S., Yoder A.M., Rautiainen R.H. (2021). Occupational Injuries among Latino/a Immigrant Cattle Feedyard Workers in the Central States Region of the United States. Int. J. Environ. Res. Public Health.

[21] Sandøe P., Anneberg I. (2019). When the Working Environment is Bad, you Take it out on the Animals—How Employees on Danish Farms Perceive Animal Welfare. https://philpapers.org/rec/SANWTW.

[22] Islam M.A. (2015). Role of Veterinarian in Animal Welfare Issue: A Global Concept. Bangladesh J. Vet. Med..

[23] Abood S.K. (2008). Effectively Communicating with Your Clients. Top. Companion Anim. Med..

[24] Artemiou E., Adams C.L., Hecker K.G., Vallevand A., Violato C., Coe J.B. (2014). Standardised clients as assessors in a veterinary communication OSCE: A reliability and validity study. Vet. Rec..

[25] Landau R.E. (2013). Assessing the Preparedness of the Veterinary Profession to Communicate with Limited English Proficient Spanish-speaking Pet Owners-ProQuest. Ph.D. Thesis.

[26] Zeller S.K., Frye M.A., Frey D.M. (2023). Spanish for Veterinarians Part 1: An Approach to Weaving Spanish Language Education into DVM Curricula. J. Vet. Med. Educ..

[27] Tayce J.D., Burnham S., Mays G., Robles J.C., Brightsmith D.J., Fajt V.R., Posey D. (2016). Developing Cultural Competence through the Introduction of Medical Spanish into the Veterinary Curriculum. J. Vet. Med. Educ..

[28] Martinez Aguiriano A.J., Salazar L., Pietrosemoli S., Schmidt M., Awosile B., Garcia A. (2024). Understanding Communication Barriers: Demographic Variables and Language Needs in the Interaction between English-Speaking Animal Professionals and Spanish-Speaking Animal Caretakers. Animals.

[29] Buendía Cambronero M. (2013). Considering cultural content in Language for Specific Purposes: Business Spanish curricula. Rech. Prat. Pédagogiques En Lang. Cah. Apliut.

[30] Ortega P., Pérez N., Robles B., Turmelle Y., Acosta D. (2019). Teaching Medical Spanish to Improve Population Health: Evidence for Incorporating Language Education and Assessment in U.S. Medical Schools. Health Equity.

[31] Petrović A. Spanish for Specific Purposes: Tourism. Proceedings of the International Scientific Conference-Synthesis 2015.

[32] Levesque D.L., Arif A.A., Shen J. (2012). Effectiveness of Pesticide Safety Training and Knowledge About Pesticide Exposure Among Hispanic Farmworkers. J. Occup. Environ. Med..

[33] Haldane S., Hinchcliff K., Mansell P., Baik C. (2017). Expectations of Graduate Communication Skills in Professional Veterinary Practice. J. Vet. Med. Educ..

[34] Pearce D. (2018). English for (Very) Specific Purposes: An ESL Course for Spanish Speaking Agricultural Workers in the Niagara Region.

[35] De Vere R. (2022). Animal welfare education and communication. Routledge Handbook of Animal Welfare.

[36] Gammelgaard J., Franzel S., Kumar A., Davis K., Preissing J., Salcedo du Bois R., Pankowska K. (2023). How to Invest in Farmers?: A Guide for Agriculture Human Capital Investment Projects.

[37] Corzo A. (2009). Spanish poultry education: Assisting the needs of the poultry industry. Poult. Sci..

[38] Garcia-Pabon J., Ostrom M. (2015). Communicating with Latino Farmers: Cultural Aspects and Strategies.

[39] Lamino P., Ceme Vinces R., Acevedo León N.F., Boren-Alpízar A., Schmidt M., McGlone J.J., Garcia A. (2025). Overcoming barriers and understanding the psychological impact of timely pig euthanasia on Spanish-speaking swine caretakers in the United States. Front. Vet. Sci..

[40] Wagner B.K., Garcia A., Calvo-Lorenzo M., Pairis-Garcia M.D. (2022). Spanish-speaking individuals within the U.S. dairy industry: Challenges and opportunities. CABI Rev..

[41] Salazar L., Martinez Aguiriano A.J., Pietrosemoli S., Garcia A. (2024). Developing Courses of Spanish for Specific Purposes in Agriculture to Bridge the Communication Gap Between the Hispanic Workforce and English-Speaking Veterinary and Animal Sciences Students. Animals.

[42] Kolb D.A. (2014). Experiential Learning: Experience as the Source of Learning and Development.

[43] Jabeen S.S. (2014). Implementation of Communicative Approach. Engl. Lang. Teach..

[44] Sayera A. (2019). The Communicative approach in English language teaching. Бюллетень Науки И Практики.

[45] Tarnopolsky O. (2012). Constructivist Blended Learning Approach: To Teaching English for Specific Purposes.

[46] Pork Checkoff Common Swine Industry Audit.

[47] American Humane Farm Program (2020). Animal Welfare Standards for Dairy Cattle.

[48] Clarke V., Braun V. (2013). Teaching thematic analysis: Overcoming challenges and developing strategies for effective learning. Psychologist.

[49] Roberts K., Dowell A., Nie J.-B. (2019). Attempting rigour and replicability in thematic analysis of qualitative research data; a case study of codebook development. BMC Med. Res. Methodol..

[50] Maguire M., Delahunt B. (2017). Doing a thematic analysis: A practical, step-by-step guide for learning and teaching scholars. Irel. J. High. Educ..

[51] Cohen J. (1960). A Coefficient of Agreement for Nominal Scales. Educ. Psychol. Meas..

[52] Burla L., Knierim B., Barth J., Liewald K., Duetz M., Abel T. (2008). From text to codings: Intercoder reliability assessment in qualitative content analysis. Nurs. Res..

[53] Landis J.R., Koch G.G. (1977). The Measurement of Observer Agreement for Categorical Data. Biometrics.

[54] Hernández Romero M.A. (2012). Nothing to Learn? Labor Learning in California’s Farmwork. Anthropol. Work Rev..

[55] Gordon H., Ramirez G., Harwell E.L., Bloss J.E., Gámez R., LePrevost C.E. (2024). Exploring the learning preferences of farmworker-serving community health workers. Health Inf. Libr. J..

[56] Maggino F. (2015). Subjective well-being and subjective aspects of well-being: Methodology and theory. Riv. Internazionale Sci. Soc..

[57] Tessmer M., Richey R.C. (1997). The role of context in learning and instructional design. Educ. Technol. Res. Dev..

[58] Vanhonacker F., Verbeke W., Van Poucke E., Tuyttens F.A.M. (2008). Do citizens and farmers interpret the concept of farm animal welfare differently?. Livest. Sci..

[59] Burns R.B. (2020). Adult Learner at Work: The Challenges of Lifelong Education in the New Millenium.

[60] Merriam S.B., Baumgartner L.M. (2020). Learning in Adulthood: A Comprehensive Guide.

[61] Dunning D., Olson J.M., Zanna M.P. (2011). Chapter five—The Dunning–Kruger Effect: On Being Ignorant of One’s Own Ignorance. Advances in Experimental Social Psychology.

[62] Eckert E. (2003). Proficiency-Development Spirals: Occupational Learning Among Farmers. Ph.D. Thesis.

[63] Alarcón L.V., Allepuz A., Mateu E. (2021). Biosecurity in pig farms: A review. Porc. Health Manag..

[64] Descovich K., Li X., Sinclair M., Wang Y., Phillips C.J.C. (2019). The Effect of Animal Welfare Training on the Knowledge and Attitudes of Abattoir Stakeholders in China. Animals.

[65] Galusha J.M. (1998). The Role of Subject Matter in Adult Learning. Ph.D. Thesis.

[66] Knowles S.M., Holton E.F., Swanson R.A. (2015). The Adult Learner: The Definitive Classic in Adult Education and Human Resource Development..

[67] Nguyen H.T., Quandt S.A., Summers P., Morgan T.M., Chen H., Walker F.O., Howard T.D., Galván L., Arcury T.A. (2015). Learning Ability as a Function of Practice: Does It Apply to Farmworkers?. J. Occup. Environ. Med..

[68] Bailey B., Lindner J., Parr B. (2017). An Examination of Georgia Young Farmer Program Participants’ Learning Style Preferences. J. Hum. Sci. Ext..

[69] Bouchrika I. (2021). The Andragogy Approach: Knowles’ Adult Learning Theory Principles for 2025. https://research.com/education/the-andragogy-approach.

[70] Little D., Green D.A. (2022). Credibility in educational development: Trustworthiness, expertise, and identification. High. Educ. Res. Dev..

[71] Jamieson K.H., McNutt M., Kiermer V., Sever R. (2019). Signaling the trustworthiness of science. Proc. Natl. Acad. Sci. USA.

[72] Augenstein I. (2021). Determining the Credibility of Science Communication. arXiv.

[73] Lamanna M., Muca E., Buonaiuto G., Formigoni A., Cavallini D. (2025). From posts to practice: Instagram’s role in veterinary dairy cow nutrition education—How does the audience interact and apply knowledge? A survey study. J. Dairy Sci..

